# Interventional Effect of Zinc Oxide Nanoparticles with *Zea mays* L. Plants When Compensating Irrigation Using Saline Water

**DOI:** 10.3390/nano14161341

**Published:** 2024-08-13

**Authors:** Mostafa Ahmed, Diaa Attia Marrez, Roquia Rizk, Donia Abdul-Hamid, Zoltán Tóth, Kincső Decsi

**Affiliations:** 1 Festetics Doctoral School, Institute of Agronomy, Georgikon Campus, Hungarian University of Agriculture and Life Sciences, 8360 Keszthely, Hungary; mostafa.ahmed.abdelmagid@agr.cu.edu.eg; 2 Department of Agricultural Biochemistry, Faculty of Agriculture, Cairo University, Giza 12613, Egypt; roquiaibrahim@gmail.com; 3 Food Toxicology and Contaminants Department, National Research Centre, Dokki, Cairo 12622, Egypt; diaamm80@hotmail.com; 4 Institute of Agronomy, Georgikon Campus, Hungarian University of Agriculture and Life Sciences, 8360 Keszthely, Hungary; szaszkone.decsi.eva.kincso@uni-mate.hu; 5 Heavy Metals Department, Central Laboratory for The Analysis of Pesticides and Heavy Metals in Food (QCAP), Dokki, Cairo 12311, Egypt; donia.atalah11@gmail.com

**Keywords:** zinc oxide NPs, maize, salinity stress, environmental stress, fatty acids, biochemical parameters, crop productivity

## Abstract

High salinity reduces agriculture production and quality, negatively affecting the global economy. Zinc oxide nanoparticles (ZnO-NPs) enhance plant metabolism and abiotic stress tolerance. This study investigated the effects of 2 g/L foliar Zinc oxide NPs on *Zea mays* L. plants to ameliorate 150 mM NaCl-induced salt stress. After precipitation, ZnO-NPs were examined by UV–visible spectroscopy, transmission electron microscopy, scanning transmission electron microscopy, energy dispersive X-ray, and particle size distribution. This study examined plant height, stem diameter (width), area of leaves, chlorophyll levels, hydrolyzable sugars, free amino acids, protein, proline, hydrogen peroxide, and malondialdehyde. Gas chromatographic analysis quantified long-chain fatty acids, and following harvest, leaves, stalks, cobs, seeds, and seeds per row were weighed. The leaves’ acid and neutral detergent fibers were measured along with the seeds’ starch, fat, and protein. Plant growth and chlorophyll concentration decreased under salt stress. All treatments showed significant changes in maize plant growth and development after applying zinc oxide NPs. ZnO-NPs increased chlorophyll and lowered stress. ZnO-NPs enhanced the ability of maize plants to withstand the adverse conditions of saline soils or low-quality irrigation water. This field study investigated the effect of zinc oxide nanoparticles on maize plant leaves when saline water is utilized for growth season water. This study also examined how this foliar treatment affected plant biochemistry, morphology, fatty acid synthesis, and crop production when NaCl is present and when it is not.

## 1. Introduction

The presence of high salt levels in the soil or water has been found to result in significant decreases in agricultural yield, ultimately leading to reduced productivity of the crops [1]. The decrease in crop yield caused by high salinity levels results from various physiological malfunctions in plants grown under saline conditions. These dysfunctions have been extensively documented in reliable reviews on the effects of salinity and plant mechanisms of defense against it [2,3]. For several decades, scientists have been competing to find solutions to the issue of salinity by implementing a range of tactics. An efficient and economical method being employed is enhancing the salt tolerance of crops. This is achieved through the use of many sorts of chemicals, including regulatory agents for plant growth, osmoprotectants, and inorganic nutrients. This approach efficiently and effectively increases crop salinity tolerance [4]. Utilizing these chemicals has significantly augmented the growth and productivity of numerous crops cultivated in saline environments [5].

Maize, scientifically known as *Zea mays* L., is a versatile crop that can thrive in various agro-climatic conditions. It is cultivated in various regions worldwide, reaching elevations up to 3000 m above sea level [6]. Farmers like this crop because it has the largest grain production potential among cereals [6,7]. It can be used for both grain and fodder [8,9], and is also grown as a cash crop for specialized corn varieties such as green ear, baby corn, sweet corn, and popcorn [10]. Additionally, it serves as a raw material for various industries. Maize is categorized as an industrial rather than a food crop since only a small fraction (12–13%) of its worldwide output is used for human consumption. The three primary staple cereals in the world, wheat, rice, and maize, make up a significant portion of the human diet. Approximately 42% of the world’s caloric intake and 37% of its protein come from them [11]. Maize is cultivated in regions with precipitation levels ranging from 300 to 500 mm, close to or below the crucial threshold for achieving a satisfactory yield [12].

In recent times, nanotechnology has become a widely recognized and effective method for reducing both biotic and abiotic stress in plants, while also promoting a sustainable approach to agriculture [13]. Moreover, it exerts a significant impact on various scientific fields such as medicine, pharmaceuticals, microbiology, and biotechnology [14,15,16,17]. Nanoparticles (NPs) have been found to have a beneficial effect on plant growth. Nanoparticles (NPs) can gather minerals at a level within cells, stimulating plant growth and development. Additionally, NPs can enhance the plant’s response to various environmental stresses and improve the supply of essential nutrients. Ultimately, this leads to higher crop yields. ZnO-NPs have garnered significant interest from scientists due to their crucial value and numerous commercial applications [18]. ZnO-NPs application alleviates several types of severe environmental stresses, such as salinity stress, enhances phenolics mobilization, chlorophyll content, and growth attributes, and stimulates the production of some mitigatory bioactive chemicals. Zn oxide nanoparticles (ZnO-NPs) have garnered significant interest from scientists due to their crucial value and numerous commercial applications. These applications ultimately improve crop yield and promote sustainable agriculture [19,20].

The present open-field investigation was carried out to study the confrontational behavior of ZnO nanoparticles on the leaves of maize plants when saline water is used to compensate for the water requirements during the growing season and how that foliar application affects the biochemistry, morphology, formation of fatty acids, and crop yield in the plants, both when NaCl is present and when it is not.

## 2. Materials and Methods

### 2.1. Layout, Scheduling, and Implements for Growing Maize Plants

**Figure A1:**
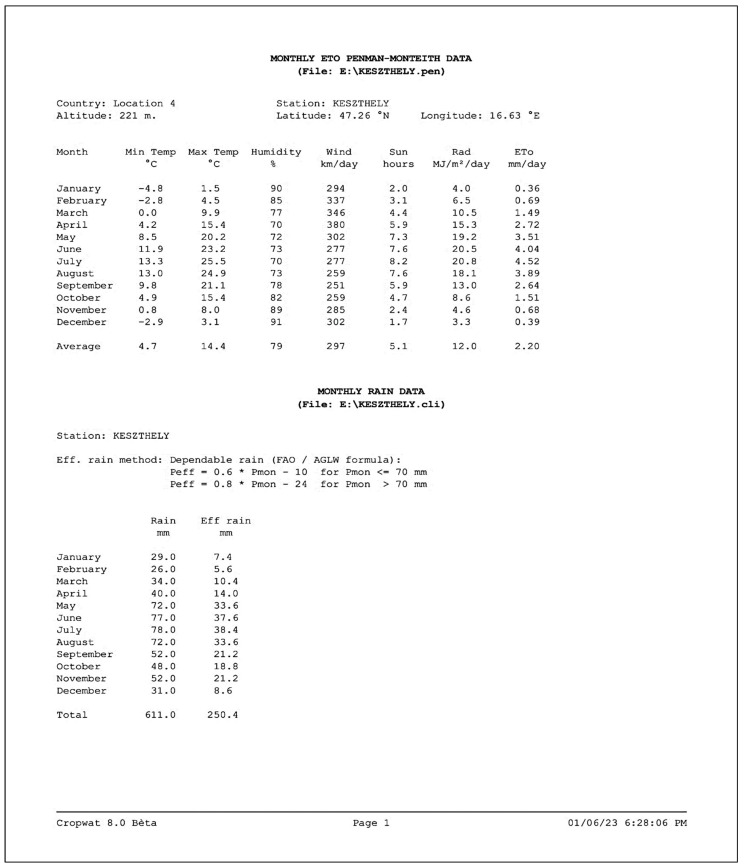
Monthly rain data using CROPWAT and CLIMWAT software.

**Figure A2:**
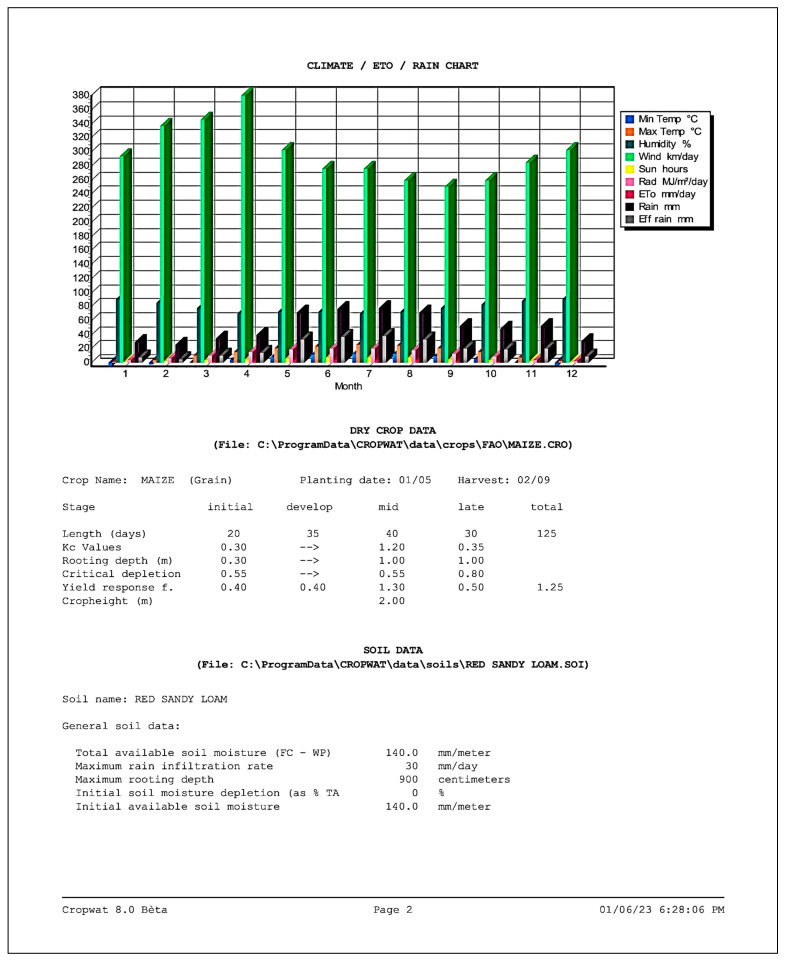
Dry crop and soil data.

**Figure A3:**
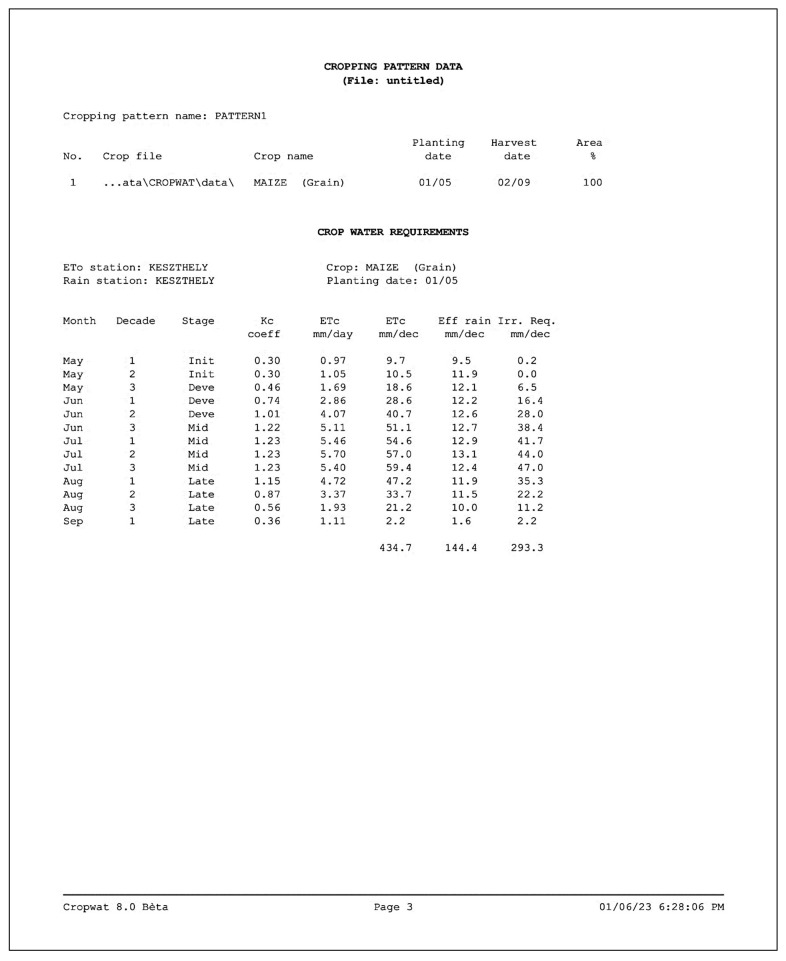
Crop water requirements.

**Figure A4:**
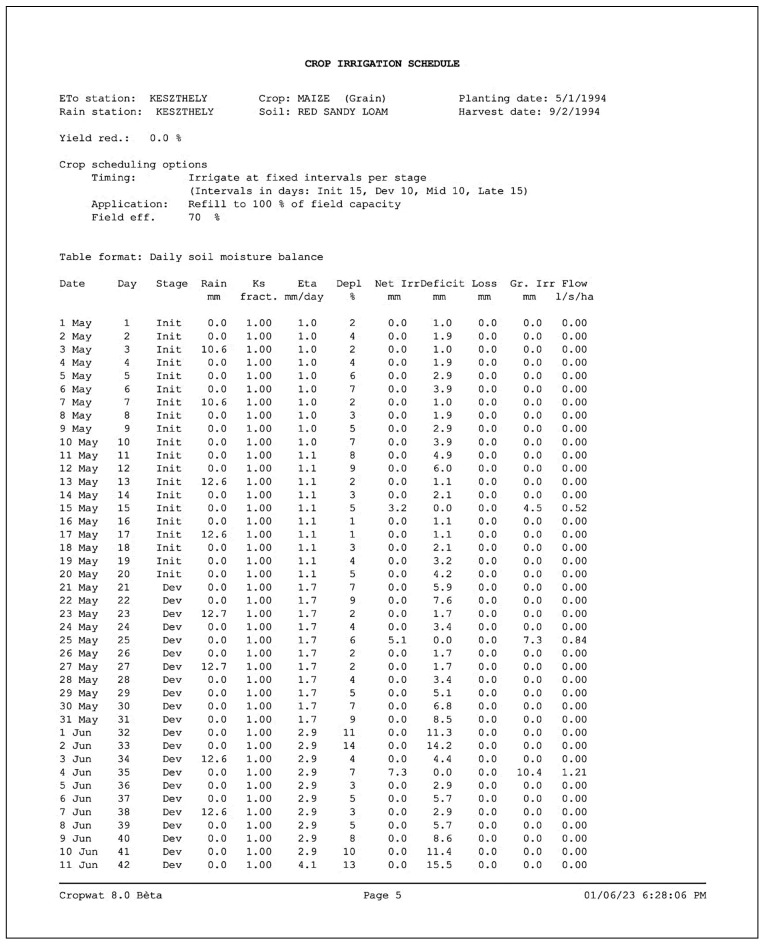

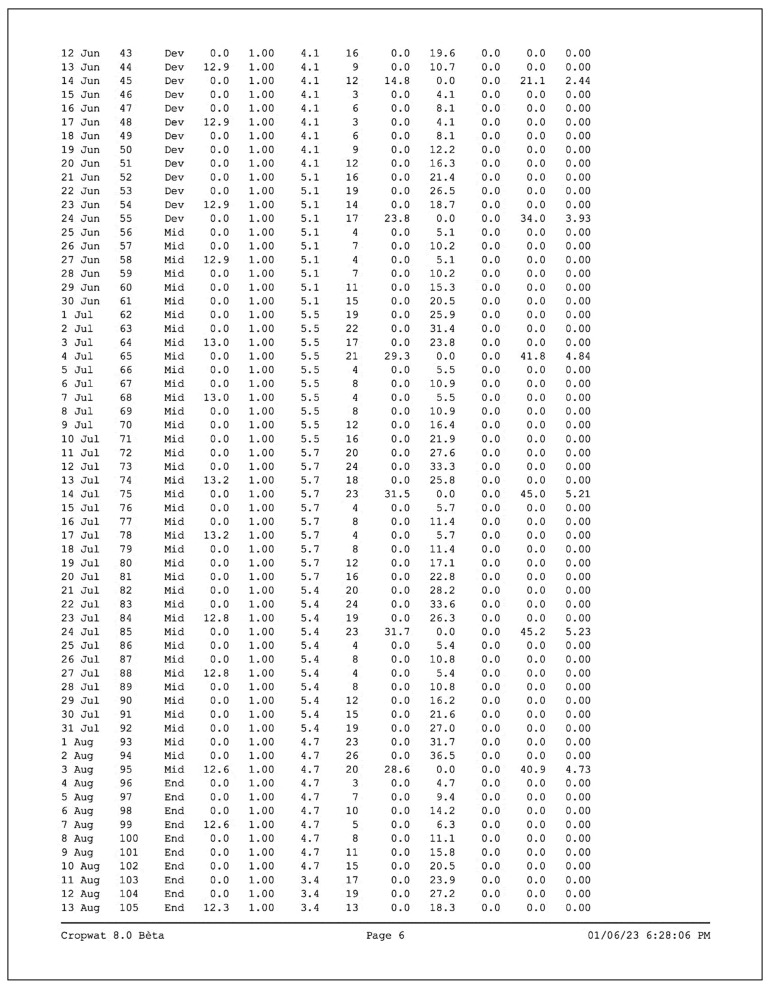

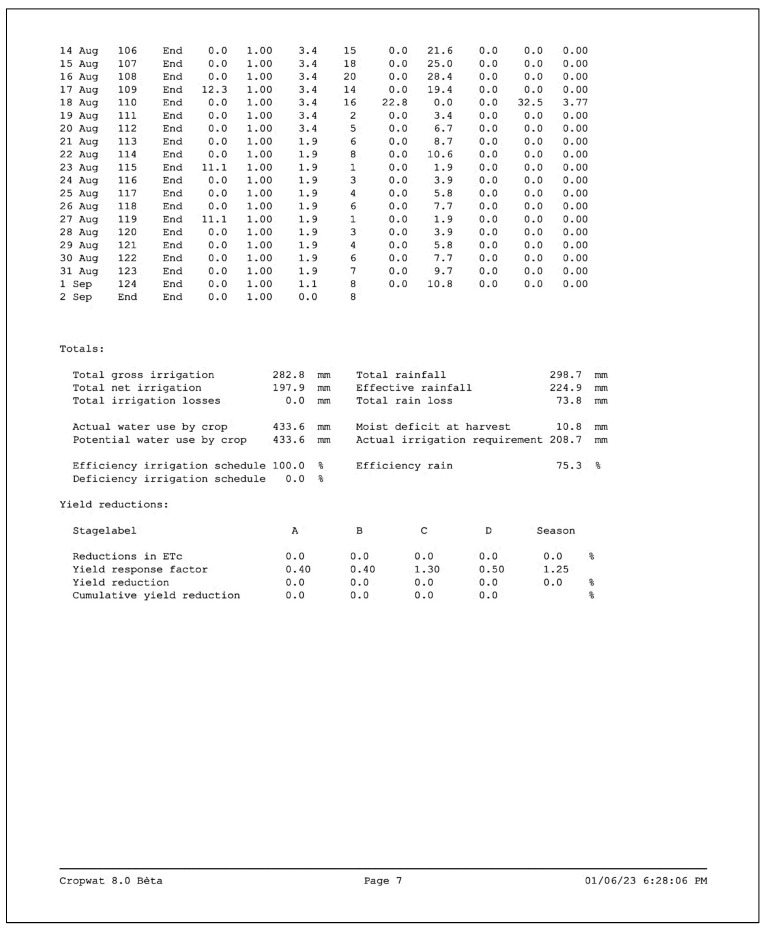
Crop irrigation schedule.

An experimental study employed a specific type of maize, FAO P0023 *Zea mays*, on soil with a sandy loam texture. The soil used had the following physicochemical properties: water percentage at a field capacity of 30.28%, water percentage at the constant point of wilting of 24.4%, bulk density of 1.2 g cm^−3^, pH of 7, with an organic matter content of 1.2%, and electric conductivity (EC) of 0.5 dS m^−1^ in the top 0.2 m of soil. The irrigation water quality employed for the experiment was satisfactory, with an electrical conductivity (EC) of 0.52 dS m^−1^ and a pH of 7.1. We conducted weekly monitoring of the electrical conductivity (EC) of the soil. The maize kernels were planted in rows within the plot, each measuring 3.0 m by 1.5 m. Each plot contained 45 seeded plants, which served as a copy. The space between two consecutive plants in the same row was 20 cm, while the spacing between two neighboring rows was 75 cm. The treatment units were organized in a fully randomized fashion with four repetitions. The plants were irrigated every 10 days. After sowing, the seeds, then seedlings, were left in the rainwater for the first 35 days to establish roots before the stress application. Salinity treatments were started 35 days after sowing (DAS), and ZnO-NPs were started at 60 DAS. The treatments were: (1) control treatment, irrigation only with tap water, (2) irrigation with saline solution (150 mM sodium chloride solution), (3) irrigation with 150 mM sodium chloride solution + 2 g/L ZnO-NPs, and (4) irrigation with tap water +2 g/L ZnO-NPs. Foliar ZnO-NPs were applied using a handheld aerosol-propelled sprayer. The salinity treatment was devised by incorporating a suitable quantity of NaCl into the irrigation water. The cumulative amount of water given from the start of planting treatments till the harvest was determined as illustrated in Table A1 and the captured figures (Figure A1, Figure A2, Figure A3 and Figure A4) from the FAO CROPWAT 0.8 and CLIMWAT 0.2 software, “https://www.fao.org/land-water/databases-and-software/cropwat/en/”, accessed on 4 June 2023. [21]. The Land and Water Development Division of FAO created CROPWAT is a decision-support tool that was used. The Land and Water Development Division of FAO designed this software application to calculate the water required for crops and irrigation. It uses data on soil, climate, and crop characteristics to make accurate calculations. Furthermore, the application facilitates the creation of irrigation schedules tailored to various management situations, as well as the computation of water supply schemes for diverse crop patterns. Farmers can utilize CROPWAT 8.0 to assess their irrigation techniques and predict crop productivity in both rainfed and irrigated environments. The previous facts reinforced the section on materials and methods, validating the techniques employed. All calculation procedures used in CROPWAT 8.0 are based on the two FAO publications of the Irrigation and Drainage Series, namely “Crop Evapotranspiration—Guidelines for computing crop water requirements”, No. 56 “https://www.fao.org/docrep/X0490E/X0490E00.htm” accessed on 4 June 2023, and No. 33 titled “Yield response to water”. The table demonstrates the volume of required water applied in the unit of liter, as the FAO CROPWAT and CLIMWAT software presented all the data in millimeters. The irrigation was applied in 9 sequential irrigations. The same water quantity of the sixth irrigation was applied for the last three irrigations. Plants were harvested 142 days after sowing. The post-harvest measurements were applied using two different plants from each replicate. The main purpose of that system of irrigation was to study the effect of compensating the irrigation water requirements of corn plants during the growing season by adding a sodium chloride solution (salt water), in combining manner of rainwater and sodium chloride solution to irrigate the corn plants. By planting, extra mono-potassium phosphate was mixed into the soil. The plants without stress were sprayed with the same amount of water. When planting began, all plots received the identical NPK fertilizers. Three instances of hand weeding characterized the entire trial period. Five plants in one of the three rows were labeled to investigate various biochemical and growth properties at the appropriate stage; in the other three rows, the plants were cultivated to maturity to evaluate grain production. Four distinct times were selected to provide the ZnO-NPs treatment to the maize plants, taking into account their vegetative and generative phases:

- 1st time, in the vegetative phase (60 DAS).
- 2nd time, in the vegetative phase (71 DAS).
- 3rd time, in the reproductive development stages (87 DAS).
- 4th time, by the end of the generative phase (100 DAS).

### 2.2. Synthesis and Analysis of the Characteristics of Zinc Oxide NPs

Following the same approach as the previous research we conducted, this study used the chemically produced ZnO-NPs [19]. The aqueous zinc nitrate and sodium hydrate solutions were mixed to create ZnO nanoparticles. ZnO nanoparticles were produced when hydroxide ions reacted with Zn^2+^ [22]. The aqueous solutions of NaOH and Zn(NO_3_)_2_·6H_2_O were mixed. The next step was to employ ultrasonic waves for 10 min to dissolve the acquired white crystals in distilled water. This would produce the ZnO-nanoparticles solution that could be applied topically. The zinc oxide NPs synthesized using the precipitation method underwent characterization through various techniques, including UV–visible spectroscopy, transmission and scanning transmission electron microscopy, energy dispersive X-ray, zeta potential, and particle size analyses as described by Ahmed et al. (2024a) [19].

### 2.3. Analysis of Chlorophyll Concentration and Morphological Characteristics

The chlorophyll concentration was measured using the soil plant analysis development (SPAD) 502Plus device manufactured by Konica Minolta (Osaka, Japan). The measurements were documented in SPAD units. The values were measured at the apical, middle, and basal regions of the most giant leaf, where the leaf collar was visible. The leaf blade was included as the surface measurement area, but the leaf vein and mid-rib were excluded. The measurements were documented at intervals of 10 days, commencing from 35 days after sowing (DAS). The leaf’s area (cm^2^) was measured using the method described by Sakalova (1979) [23]. The data were obtained by applying the following equation: S = L × W × N × K, representing the leaf surface area of the plant, measured in square centimeters (cm^2^). L represents the maximum length of a leaf, measured in centimeters. W represents the greatest width of a leaf, measured in centimeters. N represents the number of leaves in the plant at the estimation time. The correction coefficient for the maize surface was 0.75, according to Musa et al. (2016) [24]. From the ground level to the very top of the arch of the topmost leaf, with its pointed tip pointing downward, the height of each plant was measured in cm. The diameter of each plant’s stem was measured in centimeters employing the caliber.

### 2.4. Determination of Proline Content

The leaf samples, weighing 500 mg when fresh, were subjected to extraction using the solutions of C_7_H_6_O_6_S (sulfosalicylic acid), the same amount of glacial CH_3_COOH (acetic acid), and acidic ninhydrin. They were included in the extraction process. The samples were heated at a temperature of 100 °C, after which 4 mL of toluene was introduced. The light absorbed by the aspirated layer was measured at 520 nm using a Genesys UV–VIS spectrophotometer (PG Instruments Ltd., T 80, Lutterworth, UK). The proline level was quantified as milligrams per gram (mg g^−1^) of fresh weight (FW), which refers to the weight of leaves without drying or processing [19,25].

### 2.5. Protein Content Analysis

An amount of 1 g of freshly harvested leaves was homogenized in a buffer consisting of tris-HCl (10 mM, pH 8.1), HSCH_2_CH_2_OH (β-mercaptoethanol) (5 mM), C_7_H_7_FO_2_S (phenyl methane sulfonyl fluoride; PMSF; 0.57 mM), and ethylene diamine tetra acetic acid (10 mM, pH 8). The homogenization was performed using a pestle and mortar. Subsequently, the combination underwent centrifugation at 12,000 rpm/10 min. The aqueous component was collected, and Bradford’s reagent was employed to elicit the development of color. A Genesys UV–VIS spectrophotometer was used to measure the color intensity, and the protein concentration was represented as milligrams/gram fresh weight [19,26].

### 2.6. Quantification of the Concentration of Unbound Amino Acids

A fine powder was made from the fresh leaves by crushing them with a pestle and mortar. A 0.05 M phosphate buffer solution (PBS) was added to 10 mL of the resulting powder. The buffer solution’s pH was lowered to 7.8. The material that had been ground was spun in a centrifuge at 4 °C/10 min at 10,000 rpm. Half a milliliter of the extract, half a milliliter of 4% ninhydrin, and half a milliliter of 2% pyridine made up the reaction mixture in a test tube. The reaction mixture was robustly stirred in test tubes using a vortex mixer. The test tubes were subjected to 100 °C in a boiling water bath for 30 min. Utilizing a Genesys UV–VIS spectrophotometer, and the OD at 570 nm was ascertained [19].

### 2.7. Determination of Total Soluble (Hydrolyzable) Sugars

The soluble sugars were obtained by immersing dry tissue in HCl with a normal concentration of 2.5 N for 3 h in boiling water while stirring constantly. The boiling tube containing the digested sample was cooled to RT and neutralized using solid sodium carbonate until the bubbling stopped. The sample was vigorously centrifuged at 5000 rpm. To test the soluble carbohydrates, the liquid remaining after a substance has settled was completely evaporated and then mixed with a specific type of purified water. Total soluble sugars were quantified by subjecting the extract to a boiling water bath for 10 min after adding 3.0 mL of newly produced anthrone, followed by chilling. A Genesys UV–VIS spectrophotometer was used to measure the absorbance at 630 nm [19,27].

### 2.8. Assessment of Malondialdehyde (MDA)

Lipid peroxidation was quantified by assessing the concentration of malondialdehyde (MDA) using a slightly modified procedure. 0.1 g of fresh plant materials was mixed in a solution containing 0.1% trichloroacetic acid (TCA) by weight/volume. The tubes were vigorously agitated to ensure thorough mixing and then subjected to centrifugal force at a speed of 13,000 rpm (at a temperature of 4 °C) for 10 min. To prevent the Falcon tubes from bursting due to heat pressure, tiny holes were made in the cap using a syringe needle. After centrifugation, approximately 800 µL of the remaining liquid was combined with 2 mL of a solution containing 0.5% C_4_H_4_N_2_O_2_S (TBA) in a Falcon tube. The mixture was heated to 80 °C in a water bath and kept at that temperature for 25 min. The tubes were cooled on ice for 5 min and then subjected to centrifugation at a speed of 13,500 rpm (at a temperature of 4 °C) for 5 min to separate and collect any leftover TBA. The optical density was quantified using spectrophotometry at 532 and 600 nm, using a Genesys ultraviolet–visible spectrophotometer [19,28].

### 2.9. Determination of Hydrogen Peroxide (H_2_O_2_)

The recently harvested leaves, with an estimated weight of 0.5 g, were thoroughly crushed in a solution consisting of 5 milliliters containing 0.1% (weight/weight) CCl_3_COOH (TCA). After that, the mixture was subjected to centrifugation at a speed of 12,000 rpm/15 min. A solution containing a phosphate buffer PBS) (100 mM, pH 7.2), along with KI (potassium iodide), was added to a 0.5 mL reaction mixture. The vortex of this mixture was created by employing a Genesys ultraviolet–visible spectrophotometer, which quantified the light absorbed by the mixture at 390 nm [19,29].

### 2.10. Fatty Acids Profile; Gas Liquid Chromatography (GLC) Analysis

The process of extracting fat was carried out utilizing the “cold” approach by Cequier-Sánchez et al. (2008) [30]. The samples were pulverized and subjected to extraction using a combination of dichloromethane and methanol in a ratio of 2:1 (volume/volume). Subsequently, the substance underwent filtration and was transferred to a fresh test tube. An aqueous potassium chloride solution (0.88%) was introduced. Subsequently, the samples were centrifugated at a speed of 1500 rpm, and the rotary evaporator was used to evaporate the bottom layer. The standard referred to EN ISO 12966-1:2014/AC:2015; Guidelines on Modern Gas Chromatography of Fatty Acid Methyl Esters [31]. It was followed for the sample preparation of fatty acid composition determination [32]. To accomplish the above objective, 100 mg of lipids was placed into a test tube and heated on a heating block. Subsequently, the boiling mixture was supplemented with the following reagents; The substances mentioned include 2 M sodium hydroxide, boron trifluoride complex, isooctane, and a 1% sodium chloride solution. After the operation concluded, the upper layer of isooctane was transferred to a vial and subjected to analysis. Gas chromatography was employed to analyze fatty acid esters using a TRACE 2000 gas chromatograph (Thermo Scientific, Waltham, MA, USA). Fatty acids were identified using 37 standards.

### 2.11. Using NIRS™ DS 2500 FOSS to Assess Dry Matter, Protein, Neutral and Acid Detergent Fibers (NDF and ADF), and Ash in Maize Leaves and Assess the Moisture Content, Crude Protein, Crude Fat, Crude Ash, and Starch in Maize Grains (Seeds)

The Fresh Grass Silage measuring program is offered for the FOSS NIRS^TM^ DS 2500 device (FOSS Analytical A/S Co., Ltd., Whitworth Court, Manor Park, Runcorn, Cheshire, UK). It was initially designed for immediate nutrient testing after sampling corn silages, but later, it was also recommended for testing fresh samples of other silage grasses. The five parameters mentioned (dry matter content, crude protein, ash content, NDF, and ADF) were based on the water content of the plant sample sensing. The calibrations have been stabilized for sample temperatures between 10–30 °C/50–86 °F. The sample temperature was as close to the ambient temperature as possible for optimum performance. Analyzing at a temperature higher or lower than the environment temperature increases the risk for moisture condensation or evaporation from the sample and temperature drift during the analysis. NDF and ADF were determined with heat-stable amylase according to Van Soest et al. (1991) [33]. The dry matter was estimated according to the Association of Official Agricultural Chemists (A.O.A.C.); A.O.A.C. 930.15; for the crude protein in leaves, A.O.A.C. 990.03 was used, and ash was analyzed according to A.O.A.C. 923.03 [34]. Crude fat, ash, and protein contents in the grains were estimated according to Van Soest et al. (1991) as well [33].

### 2.12. Statistical Analysis

This study’s experimental methods included completing each evaluation four times. The average value and standard error of the data were then given. The Central Coastal Agricultural Research Institute (ICAR) used the Web Agri Stat Package (WASP) to conduct the statistical study. ANOVA (one-way) was employed in this study to examine group differences. Using significance levels of 1% and 5%, the least significant difference (LSD) test was utilized, and the results were displayed as a critical difference (CD) [35].

## 3. Results

### 3.1. Characterization and Investigation of Chemically Generated Zinc Oxide NPs

**Figure 1:**
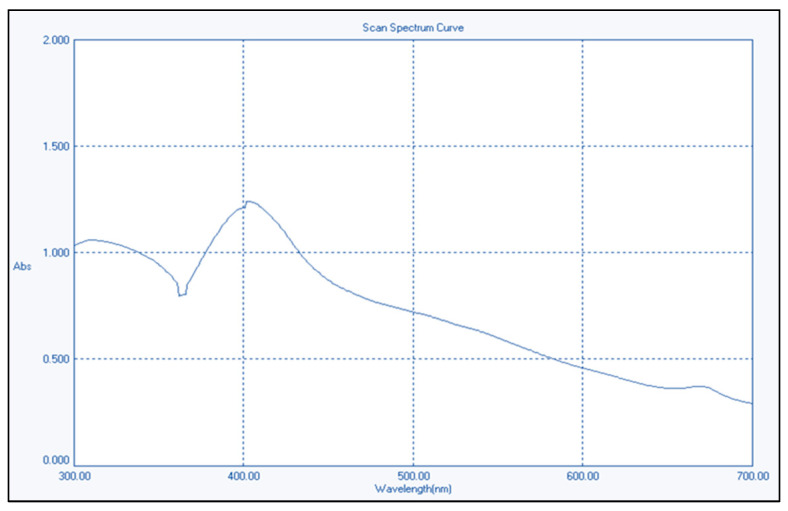
Ultraviolet–visible spectrum of chemically generated ZnO nanoparticles.

Figure 1 shows the spectrum of a distinct zinc oxide absorption peak at 403 nm, corresponding to ZnO’s intrinsic band-gap absorption. The absorption occurs due to electron shifts between the conduction and valence bands (O_2p_→Zn_3d_).

**Figure 2:**
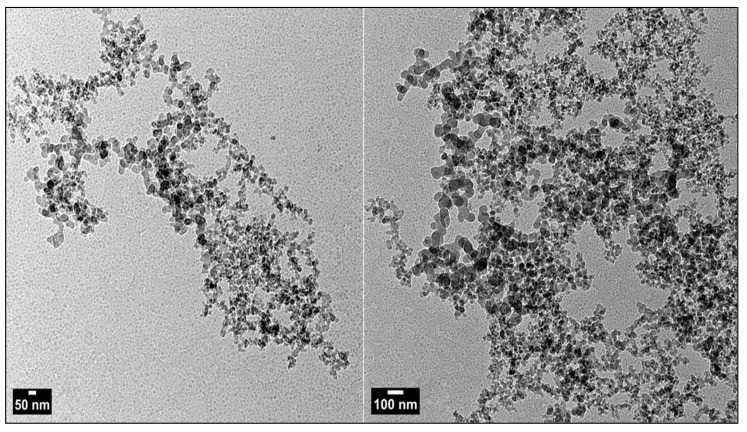
Transmission electron micrographs showing chemically generated zinc oxide NPs on substrates with sizes of 50 and 100 nanometers.

**Figure 3:**
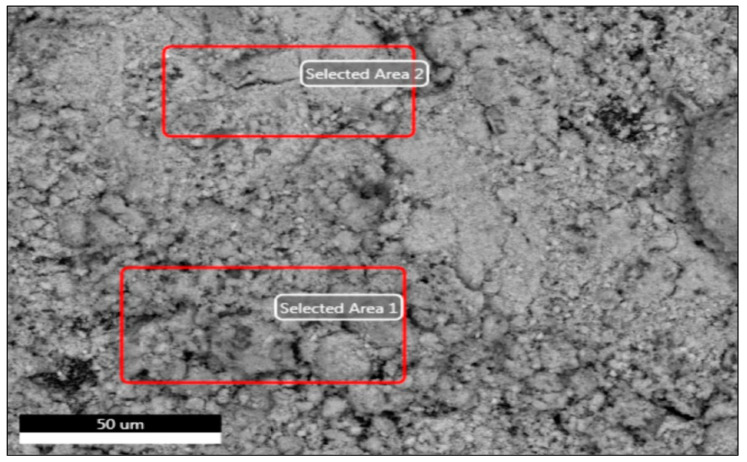
Scanning transmission electron micrographs of chemically generated zinc oxide NPs on a substrate measuring 50 microns.

**Figure 4:**
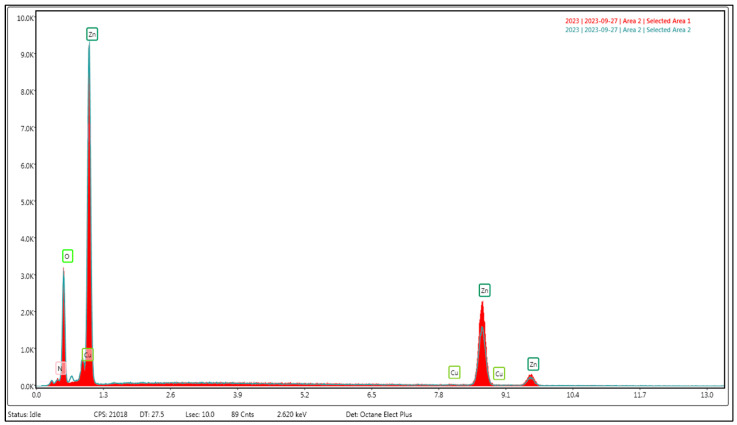
Energy dispersive X-ray spectrum of chemically generated zinc oxide NPs.

The chemically generated ZnO-NPs are examined in Figure 2, Figure 3 and Figure 4. The analysis comprises transmission and scanning transmission electron microscopy techniques, energy-dispersive X-ray spectroscopy, and size. The TEM image (Figure 2) revealed the formation of zinc oxide NPs in a near hexagon shape, indicating the high quality of the newly formed particles. Figure 3 displays the STEM pictures of the ZnO-NPs, illustrating the particles’ uniform shape and size for the particles in two specific regions. Furthermore, the presence of ZnO-NPs in the form of powder is evident. Figure 4 confirms that the primary constituents of the ZnO sample are zinc and oxygen, and these elements are evenly distributed across the surface of the zinc oxide NPs.

**Figure 5:**
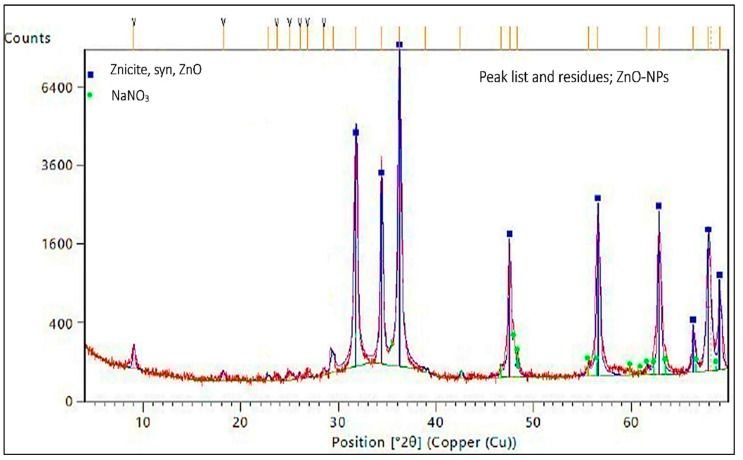
X-ray diffractometer pattern of chemically generated zinc oxide NPs.

Figure 5 exhibits the X-ray diffraction (XRD) peak list of chemically generated zinc oxide nanoparticles and the remaining remnants of sodium nitrate (NaNO_3_). The diffraction of the primary peaks for ZnO-NPs is observed in the color blue.

**Figure 6:**
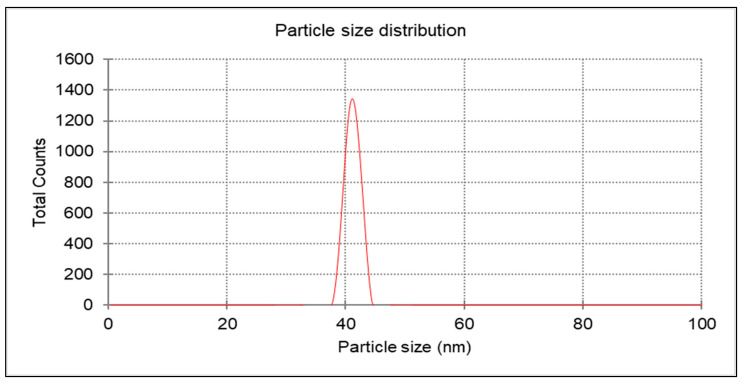
The particle size of chemically generated ZnO-NPs is used to measure their charge.

The ZnO-NPs, synthesized by precipitating at 200 °C for 2 h, have a monomodal size distribution. The distribution is approximately 41.166 nm. One measure of a collider’s potential stability is its zeta potential magnitude. This study showed that the nanoparticles were very stable, with a zeta potential of −21.4 mV [19]. Zinc oxide nanoparticles that were chemically produced had their zeta potential measured in water, which served as a dispersion. And, as shown in Figure 6, the size was found to be 41.166 nm.

### 3.2. Evaluation of the Chlorophyll and Growth Characteristics in Various Treatments of Maize

The chlorophyll levels in the various treatments were measured using soil plant analysis development (SPAD) units, as indicated in Table 1. A four-stage examination assessed the impact of NaCl stress and foliar spraying of ZnO-NPs. During the initial stage, there was no significant difference in the chlorophyll levels across the various treatments. However, the chlorophyll content dropped over time, specifically in the stressed treatment that did not receive ZnO-NPs spray (T2). Conversely, the chlorophyll levels increased in the non-first and fourth treatments (T1 and T4) and the stressed treatment that received a spray of ZnO-NPs (T3). In the fourth stage, it was observed that the stressed treatment sprayed with ZnO-VPS (T3) had the same chlorophyll content as the non-stressed treatments (control “tap water” and “tap water + ZnO-NPs 2 g/L”) (T1 and T4). The results demonstrated that spraying ZnO-NPs substantially increased chlorophyll levels in the third treatment (T3).

The height of the maize plants (measured in centimeters) under each treatment was determined and recorded in Table 2. Four stages were identified to assess the impact of chlorophyll concentration on growth and morphological characteristics during the experiment. Significant differences in maize plant heights were observed among the different treatments at all stages. The maize plants exhibited height increases in response to the four treatments, except for the second treatment (T2), where height growth ceased after the third stage. It was noted that the growth rate in the non-stressed treatments (T1 and T4) and the stressed treatment treated with ZnO-NPs, was more significant than in the stressed treatments (T2). At the fourth stage, the growth rate of maize plants’ height in the third treatment (T3) approached the elongation rate observed in the first and fourth treatments (T1 and T4) when ZnO-NPs were applied through spraying. In contrast, the rate of growth in height of maize plants was higher for the fourth treatment (T4), where no stress was applied and the plants were sprayed, compared to the control treatment (T1).

The thickness (in centimeters) of the maize stem was measured for each treatment, as indicated in Table 3. Like the previous measurement of growth and morphological characteristics (namely plant height), stem width was assessed in four phases. Significant differences in stem width of maize plants were detected across all stages in the other treatments. Until the third stage, it was noted that the growth rate in the first and fourth treatments (T1 and T4) was similar to the control treatment and more significant than the second treatment (T2). During the fourth stage, the rate of maize stem width growth was higher for the non-stressed and sprayed treatment (T4) compared to the control treatment (T1) and the third one (T3). Treatment T4 showed a slight rise in comparison to treatments T1 and T2.

The leaf area of maize (measured in cm^2^) in each treatment was determined and is presented in Table 4. The leaf area was assessed in four stages to evaluate the impact of NaCl stress and foliar application of chemically produced zinc oxide nanoparticles. The growth rate in the first and fourth treatments (T1 and T4) was similar to the control treatment and more significant than the second treatment (T2). During the fourth stage, it was noticed that the rate of maize leaf area growth was higher in the non-stressed and sprayed treatment (T4) compared to the control treatment (T1) and the treatment sprayed with ZnO-NPs (T3). This indicates that the leaf area increased faster in T4 than in T1 and T3.

### 3.3. Analysis of Maize Leaves That Were Submitted to a Variety of Treatments to Identify Several Biochemical and Stress Parameters

The results are presented in Table 5. The maize leaves’ hydrolyzable sugars, free amino acids, proline, hydrogen peroxide (H_2_O_2_), and malondialdehyde (MDA) contents all rose as the maize leaves were exposed to the salt. This study discovered that treatments’ effects on plant contents of the prior stress and biochemical markers were statistically significant. Compared to the three previous treatments (T1, T3, and T64), the second treatment (T2) had the highest concentrations of the mentioned parameters. The only exception was the protein content, which declined to the lowest content among the various treatments in the second treatment (T2). Spraying the treatments (T3 and T4) with a zinc oxide nanoparticle solution at a concentration of 2 g/L was employed. As was noted before, the secretion of the compounds was controlled to ensure that it was compatible with the initial therapy (control) (T1). The predicted parameter values for the second treatment (T2) were in the greatest range compared to those of the other treatments (T1, T3, and T4), regardless of whether they were stressed. It was observed that the concentrations of hydrolyzable sugars, total free amino acids, proline, H_2_O_2_, and MDA were as follows: 177.50 ± 5.60 mg/g, 53.83 ± 1.23, 1.56 ± 0.03, 0.22 ± 0.00 mg/g, 1.02 ± 0.01 mg/g, and 5.16 ± 0.46 mmols/mL, respectively. The highest value for protein content was found in the fourth treatment, and the concentration of 2.39 ± 0.03 mg/g was the highest value reported for this treatment. The indicators that had been previously estimated to have the lowest concentrations were 170.76 ± 2.86 mg/g of hydrolyzable sugars estimated as glucose in T4, 53.83 ± 1.23 mg/g of free amino acids determined as L-leucine in T1, 0.22 ± 0.00 and 0.23 ± 0.00 mg/g of proline in T1 and T4, respectively, 1.02 ± 0.01 mg/g of H_2_O_2_ in T1, and 5.16 ± 0.46 and 5.97 ± 0.41 mmols/mL of MDA in T1 and T4, respectively, and 0.93 ± 0.01 and 0.98 ± 0.02 mg/g of protein estimated as bovine serum albumin (BSA) in T2 and T3, respectively.

### 3.4. Finding Various Post-Harvest Parameters in Maize Seeds and Leaves from Diverse Treatments

Table 6 shows the results; the salty water-treated plants showed the lowest values of the different examined post-harvest parameters such as weight of the humid and dried, weight of the humid and dried stalk, weight of the humid cob, weight of humid and dried 100 seeds, weight of humid and dried all seeds in the cob, the moisture content in the corn seeds, and even the number of the rows and seeds per the cob. On the other hand, the foliar-sprayed plants with ZnO-NPs, even if they were stressed or not, showed higher values than in the salty-water irrigated treatments, sometimes the values were also higher than were in the control treatment.

### 3.5. Analyzing the Dry Matter, Protein, and Acid and Neutral Detergent Fibers in Maize Leaves and the Water Content, Protein, Fat, and Starch in Maize Grains (Seeds)

Table 7 shows the changes in dry matter, crude protein, ash, ADF, and NDF values in the leaves of maize. No statistically significant differences were found between the treatments according to the dry matter. However, the fourth treatment (T4) showed the highest values in crude protein content, ash, and acid and neutral detergent fiber contents compared with the other three treatments (T1, T2, and T3), showing the positive effect of ZnO-NPs foliar application. The second treatment (T2) showed the lowest values, although sometimes the values of the third treatment, which was stressed but sprayed with ZnO-NPs, showed some close values to that treatment.

The moisture, crude protein, crude fat, crude ash, and starch values of maize seeds were analyzed and are displayed in Table 8. Crude fat content did not show any statistically significant differences across the treatments. T4, which did not undergo stress but was sprayed with ZnO-NPs, exhibited the most significant ash values and crude protein content. T3, in contrast, had the greatest moisture and starch content values when compared to the other treatments.

### 3.6. Gas Chromatographic (GC) Analysis of Long-Chain Fatty Acids (LCFA) in Maize Seeds (Grains)

The long-chain fatty acid (LCFA) profile of maize seeds’ extracted oil was determined, as shown in Table 9. The prepared standards expressed 15 fatty acids. Three fatty acids were not detected in the investigated maize seeds’ extracts. These fatty acids were Eicosapentaenoic acid (EPA), docosadienoic acid, and docosapentaenoic acid. It was noticed that the obtained fatty acids from the stressed treatment that was not sprayed with ZnO-NPs (T2) were higher in concentration, with totals of 97.56 mg/g, compared with the other three treatments that showed concentrations of 94.94, 96.00, and 95.78 mg/g in T1, T3, and T4, respectively. Table 9 shows the presence myristic acid, palmitic acid, palmitoleic acid, stearic acid, oleic acid, vaccenic acid, linoleic acid, γ-linolenic acid, α-linolenic acid, ecosenoic acid, arachidonic acid, and docosahexaenoic acid (DHA).

## 4. Discussion

### 4.1. Characterization of Chemically Generated Zinc Oxide NPs

The previously synthesized chemically ZnO-NPs used in our latest research article [19], were also used in the current study in different concentrations. They were synthesized using the same precipitation method and re-characterized under the same conditions. The UV–VIS spectra exhibited the highest absorption of surface plasma resonance in the 400–500 nm range for the produced ZnO-NPs. The presence of this specific absorbance peak indicates the synthesis of ZnO-NPs. The unique range of the pure ZnO-NPs and the absence of any other peaks in the spectrum further provided additional evidence of the purification of the generated NPs and the exclusive synthesis of ZnO-NPs without any other compounds. The produced nanoparticles were analyzed using transmission electron microscopy (TEM) to accurately determine their crystalline characteristics and size. TEM images of ZnO validate the hexagonal shape of the particles and demonstrate minimal fluctuation in thickness, which is consistent with the findings of SEM. TEM pictures showed that most ZnO nanoparticles (NPs) exhibit a hexagon shape, with particle sizes ranging from 50 to 100 nanometers. The surface morphologies produced by ZnO-NPs were also analyzed using SEM. The scanning electron microscope (SEM) image of the ZnO-NPs reveals the presence of spherical structures with particles clustered closely together. The preceding findings of UV–VIS, TEM, and STEM analyses were consistent with the results demonstrated by Maher et al. (2023) [36]. The results of our latest study [19] confirmed the size of the ZnO-NPs was 41.166 nm.

The EDAX examination verified the existence of the chemically acquired ZnO-NPs. The elemental composition of the ZnO-NPs revealed the presence of zinc and oxygen, confirming that the produced ZnO-NPs are in a pure state. Using a copper grid that is lacy carbon coated increases the quality of STEM electron micrographs, which was why the EDAX analysis included copper. The crystalline nature of ZnO-NPs was evaluated using X-ray diffraction. The diffraction pattern exhibited nine prominent peaks at 2θ values. The average particle size of zinc nanoparticles was determined, which is particularly significant for the ZnO structure. The presence of observable line broadening in the XRD peaks suggests that the produced material has a size in the nanometer range. The prominent peaks indicate that the ZnO nanoparticles had a high level of crystallinity. The discovered results align with the findings of other researchers who have used the XRD diffractometer to analyze the synthesized ZnO-NPs [37]. The stability of ZnO-NPs is guaranteed by the stability shown by zeta potential. So, the zeta potential examination of the nanoparticles in this study was performed, and the result was approximately −21.4 mV, indicating that the chemically produced ZnO-NPs demonstrated excellent stability. Consistent with the findings of Amin et al. (2023), the present negative surface charge potential lends credence to dispersity and long-term stability [38].

### 4.2. Analyzing the Effects of Various Maize Treatments on Growth and Chlorophyll Levels

Exposure to salt stress severely reduced the growth and morphological traits of maize plants, including stem width, leaf area, chlorophyll levels, and plant height (150 mM NaCl) to compensate for the irrigation requirements in this study in the second treatment (T2) compared to the other treatments in all stages that the measurements were recorded in. However, spraying ZnO-NPs, particularly on the third and fourth treatments (T3 and T4), greatly enhanced the previously mentioned growth metrics by ameliorating the saline-stress-mediated decline in T3 or the positively stimulated non-saline-stressed (T4) as shown in Table 1, Table 2, Table 3 and Table 4.

The regulation of the apoplastic and symplastic pathways is crucial in alleviating the harmful effects of high salinity on the entry of Na^+^ and Cl^−^ into the transpiration stream, thus preventing ionic poisoning in the top sections of the plant [39,40]. Exceeding a specific threshold, the presence of Na^+^ and Cl^−^ ions hampers the process of protein synthesis, restricts metabolic function, impairs cellular structures, and ultimately leads to cell death [41,42]. Moreover, it stimulates the generation of reactive oxygen species (ROS), which, when they build up, cause oxidative damage to nucleic acids and the denaturation of the plasma membrane. This, in turn, impacts osmotic pressure, cellular elongation, and cell division [39,41].

The observed improvement in the performance of the third treatment plants was attributed to utilizing ZnO-NPs. ZnO-NPs treatment improves plant growth by increasing resilience to abiotic stress [43]. Using ZnO nanoparticles in plants significantly facilitates the production of plant hormones, such as indole acetic acid (IAA) and gibberellic acid (GA3), leading to increased synthesis. Consequently, this process enhances plant growth by increasing dividing cells and expansion, maintaining intact membranes, and activating enzymes. It enables plants to withstand abiotic stresses, such as salinity [44]. Zinc (Zn) nanoparticles alleviated the adverse impacts of saltwater stress by regulating metabolic activities in plant cells under stress and promoting the proper synthesis of pigments for photosynthesis. El-Badri et al. (2021) observed a notable enhancement in the length of shoots in *Brassica napus* L. when subjected to 150 mM NaCl stress after being treated with ZnO-NPs seed priming, as compared to the control group [45].

### 4.3. Analysis of Various Biochemical and Stress Indicators in Variously Treated Maize Leaves

The H_2_O_2_, MDA, proline, total free amino acids, and total hydrolyzable sugar levels significantly increased in response to salt stress [19,46] (Table 5). The maize leaves exhibited the most elevated levels of stress indicators in the second treatment, with 228.49 ± 2.17, 74.35 ± 1.19, 0.32 ± 0.03, 1.78 ± 0.02 mg/g, and 13.06 ± 1.61 mmols/mL, for sugars, free amino acids, proline, hydrogen peroxide, and malondialdehyde, respectively. After spraying with 2 g/L ZnO-NPs, the levels were decreased in the third and fourth treatments (T3 and T4). It was also noticed that the control treatments that were not stressed or sprayed with ZnO-NPs had the lowest values of those determined stress markers compared to the second stressed treatment, the sprayed stressed third treatment and even the fourth treatment that was not stressed at all but sprayed with zinc oxide NPs. The amount of protein in maize leaves was minimal in the second treatment, which involved exposure to 150 mM NaCl and no application of ZnO-NPs, and the protein level measured 0.93 ± 0.01 mg/g. However, it was clear that the protein concentrations noticeably increased in the fourth treatment (T4) and gave 2.39 ± 0.03 mg/g. But, in the case of the third treatment (T3), it gave 0.98 ± 0.02 mg/g. It was found that the protein content in cluster beans and green peas was increased by applying ZnO NPs. Furthermore, it is widely recognized that Zn is an essential element necessary for the optimal development and growth of plants. The roots’ cation-exchange ability enhances nutritional absorption, particularly nitrogen, which leads to greater protein content [47,48].

The elevation in MDA, H_2_O_2_, and other identified biomolecules in plants under salt stress can be linked to the heightened reactive oxygen species (ROS) and the consequential impairment of cell membranes. The current study demonstrated that applying zinc oxide nanoparticles to the leaves enhanced the plants’ sensitivity to NaCl stress. The preceding report can be attributed to the fact that ZnO nanoparticles enhanced plant resilience to sodium chloride stress by mitigating the generation of ROS and oxidative harm, leading to mitigating the harmful effects of salinization. The decrease in ROS generation is linked to a decline in enzymatic and non-enzymatic antioxidant activity [49,50]. Another possible explanation is the impact of ZnO-NPs on the absorption of nutrients, which counteracts the deficit of micro and macronutrients caused by NaCl in plants [51]. In some other studies and in line with the obtained results in the current experiment, evidence has demonstrated that zinc oxide NPs have increased the absorption of calcium, potassium, zinc, iron, and copper in faba bean [52] and rapeseed [45] when exposed to high salinity levels, these components replaced the Na ions, reducing the harmful consequences of Na+ toxicity.

### 4.4. Determination of Different Post-Harvest Measurements in the Leaves and Seeds from Different Treatments of Maize

Zinc oxide nanoparticles significantly enhanced the weight (humid/dried) of cob and seeds in foliar-treated plants as compared to stressed (without spraying) and control plants (Table 6). They recorded maximum cob and all seeds humid weight at 307.53 ± 3.50 g and 254.02 ± 1.06 g in 2 g/L of ZnO-NPs sprayed fourth treatment (T4) compared to salt-stressed second treatment (T2) at 211.98 ± 13.75 g, and 184.15 ± 11.78 g, respectively. Nano foliar application (2 g/L) also enhanced the weight (humid/dried) of cob and seeds in foliar-treated stressed plants (T3) compared to salt-stressed non-sprayed second treatment (T2). The number of seeds/row was also noted. In foliar-treated plants, even if they were stressed (T3) or non-stressed (T4), the maximum number of seeds/row was 43 seeds, and 41 seeds/row compared to the salt-stressed treatment that was not sprayed with ZnO-NPs and control as they recorded 36 and 40 seeds/row, respectively.

Raddy et al. (2018) examined the impact of applying zinc oxide nanoparticles to the leaves of maize plants on their development and productivity. The researchers observed an increase in both the length and weight of the cobs in plants treated with nanoparticles, compared to those treated with zinc oxide in a bulky form and the control group [53]. Tondey et al. (2021) observed improved maize yielding characteristics with seed priming with zinc oxide nanoparticles [54]. Satdev et al. (2020) reported that using zinc nanoparticles as a priming ingredient and foliar spray increased the length, girth, and number of cobs per plant [55]. The likely cause could be attributed to the high absorption and efficient movement of nano zinc oxide. The correlation may be attributed to the heightened chlorophyll levels and photosynthetic activity observed in maize plants treated with ZnO-NPs [56]. Zhou et al. (2011) further elucidated that ZnO nanoparticles (NPs) exhibit a high affinity for physical surfaces and strongly interact with biological proteins owing to their elevated specific surface reactivity. Consequently, this leads to enhanced absorption on the cellular surface. According to Lin and Xing (2008), ZnO-NPs were primarily penetrated to the cell surface and rapidly removed. The enhancement observed in plants treated with foliar application may be attributed to the swift transportation and incorporation of Zn nanoparticles, which subsequently stimulates the production of enzyme activity that accelerates growth and the metabolism of auxin in plants [57].

### 4.5. Assessment of Dry Matter, Protein, Neutral and Acid Detergent Fibers in Maize Leaves, and the Moisture Content, Protein, Fat, and Starch in Maize Grains (Seeds)

The current study showed no significant differences in the dry matter content in the different treatments. The crude protein determined using the NIRS matched the obtained results from the colorimetric technique used to determine the protein content in the leaves, as the protein values were decreased in the salt-stressed plants leaves from the second treatment (T2) compared with the plants that were sprayed with 2 g/L ZnO-NPs in the third treatment(T3), the control plants (T1), and the non-stressed plants that were only sprayed with 2 g/L ZnO-NPs (T4).

The consumption and digestibility of fodder are significantly impacted by parameters such as the number of cell walls, the ratio of tissues with different biodegradability, the composition of the chemicals, the proportion of lignin, and other physicochemical factors that affect the digestibility of cell walls in the rumen [58]. ADF and NDF data evaluate forage digestibility, total digestive nutrients, and energy and relative feed values. The relative feed value is an index that allocates forage for animal performance. The entire cellular structure, comprising ADF (acid detergent fiber) and hemicellulose, is classified as NDF (neutral detergent fiber). NDF values quantify the amount of forage an animal can consume. ADF stands for the cell walls of forage composed of cellulose and lignin. These variables impact the process of fodder digestion in animals. The assessment also includes an evaluation of prices and the management, harvest, and storage procedures for fodder [59]. The current study showed that the ADF and NDF values were decreased in the salt-stressed plants leaves in the second treatment (T2) compared with the salt-stressed plants from the third treatment that was sprayed ZnO-NPs (T3), the control plants (T1), and the non-stressed plants from the fourth treatment that were only sprayed with 2 g/L ZnO-NPs (T4). The results proved the effects of applying the foliar spray of ZnO-NPs on ADF- and NDF-enhanced values. Previously, saline stress has been linked to decreases in NDF and ADF [60], for *Lotus corniculatus* shoots and leaves, and other legumes. Higher salt concentrations in water were found to be positively correlated to a decrease in NDF level and an increase in the level of soluble carbohydrates in *Lolium multiflorum*, Lam [61]. The composition must consist of oligo- and polysaccharides, low molecular weight and cell wall carbohydrates like pectins and highly soluble hemicelluloses. Previous studies have shown that carbohydrates, such as glucose, fructose, sucrose, and starch, tend to build up when exposed to stress caused by salt [58,62,63,64].

The current study showed that the crude protein values in the grains were decreased in the leaves of the salt-stressed plants, even they were not sprayed with 2 g/L ZnO-NPs as in T2 or were sprayed with 2 g/L ZnO-NPs as in T3, compared to the non-stressed plants that were only sprayed with 2 g/L ZnO-NPs (T4), followed by the control treatment (T1). The results showed the positive effect of spraying ZnO-NPs on increasing the crude protein values in maize grains. However, there were no noticeable differences observed across the treatments for the crude fat content. For starch content, the third treatment (T3) showed the highest content of starch compared to the non-stressed treatment that was sprayed (T4), and the stressed treatment that was not sprayed (T2). The moisture content in the salt-stressed treatment (T2) was higher than in the non-stressed sprayed treatment (T4), and the control treatment (T1). However, the stressed treatment sprayed with ZnO-NPs (T3) showed the highest moisture content.

Despite varying environmental conditions that can significantly impact the quality of maize grains [65]. There is not enough investigation into the detrimental effects of salt stress on the quality of grains. While the impact of different salinity levels on the starch and protein content in maize grain yielded conflicting results, it is essential to maintain an appropriate equilibrium of salt levels in the irrigation water to control the quantity of each constituent. Optimal moisture content in the grain is advantageous for storage reasons, as it inhibits the growth of fungal infections that can lead to mycotoxin contamination and a decline in the quality of maize grain [66]. Li et al. (2019) observed no discernible variations in maize grain’s oil, crude fiber, and ash compositions. On the other hand, as salinity increased, there was a drop in grain moisture and starch content. The highest values were when the total dissolved solids (TDS) reached 1, 2, and 3 g L^−1^. Conversely, protein content increased with higher salt levels, reaching maximum values over 12% when the TDS was 4 and 5 g L^−1^ [67]. Nevertheless, the outcome did not match the findings of Cucci et al., who confirmed that there was no notable disparity in the moisture, starch, protein, and oil content of the kernel [68]. The quality of maize grain is directly related to the concentration of starch and protein, with higher levels of these nutrients indicating better quality. According to Weinberg et al., storing maize with low grain moisture content is preferable since it reduces the likelihood of fungal infections and the risk of mycotoxin contamination in the kernels [66].

### 4.6. Gas Chromatographic (GC) Analysis of Long-Chain Fatty Acids (LCFA) in Maize Grains (Seeds)

The current study revealed that the concentration of the obtained fatty acids from the second (T2) was lower than the other treatments, with totals of 97.56 mg/g, compared with the other three treatments that showed concentrations of 94.94, 96.00, and 95.78 mg/g in T1, T3, and T4, respectively. This study showed a positive correlation between exposure to salt stress and secreting the fatty acids and the mitigatory influence of ZnO-NPs on limiting the production of those secreted fatty acids as a response to salinity stress. The examination of fatty acids in corn, Triticum, groundnut, arabidopsis, and *Suaeda salsa* under salinity stress revealed that the concentrations of unsaturated FAs rose as a response to counteract the effects of salt stress [69,70]. Oleic acid, linoleic acid, and linolenic acid are the primary unsaturated fatty acids that determine the level of unsaturation in most plants. An analysis of *Vitis vinifera* seeds using lipid profiling and GC–MS/MS demonstrated that particular unsaturated fatty acids serve as precursors for the synthesis of prostaglandins and jasmonates in response to environmental stresses [69]. Salinity stress in sunflowers results in a significant decrease in linoleic acid and δ-tocopherol. At the same time, there was an increase in the levels of palmitic, stearic, linolenic acids, and α- and γ-tocopherols [71]. Recent research has demonstrated that exposure to high salt levels reduces the activity of certain enzymes called desaturases, namely the membrane-bound ω-3 and ω-6 desaturases in the olive mesocarp. As a result, this alters the ratio of oleic acid to linoleic acid [72]. Oleic acid serves as a critical stress-inducer in plants, as it stimulates phospholipase D enzyme (PLD) [73]. Liu et al.’s (2019) research on plant lipid remodeling has demonstrated that palmitic and oleic acids concentrations exhibited an increase as a consequence of abiotic stress, which matches the findings of the current research [74]. Research on lipid alteration in salt-sensitive and salt-tolerant barley roots reveals that the salt-sensitive type exhibits lower amounts of 18:2 and 18:3 lipids [75].

In the present study, the level of oleic acid was upregulated by the exposure to salinity stress in maize grains of the second treatment (T2). Increasing the oleic acid (18:1) in salt-stressed grains indicated the tolerance to salinity. As the presence of salinity stress increases, the formation of oleic and linoleic acid increases, boosting the plasma membrane’s activity. H^+^-ATPases are responsible for regulating ion homeostasis in the roots of barley plants [76]. The saline growing conditions were shown to be associated with a reduction in cis-vaccenic acid. In their study, Paulucci et al. (2011) found that there was a significant reduction in the proportion of cis-vaccenic acid and a corresponding rise in the case of saturated fatty acids in peanut nodulating rhizobia when exposed to high temperatures and salinity [77]. Gogna et al. (2020) found that the levels of cis-vaccenic acid in the 1,2-diacylglycerol fraction of the variety, which was salt sensitive, decreased significantly during salinity stress. It is worth noting that cis-vaccenic acid was not present in the salt-tolerant and semi-tolerant varieties. Therefore, cis-vaccenic acid can serve as an indicator or signal for salt-sensitive sunflower types experiencing salt stress [69]. Eicosanoic acid levels were slightly elevated under salinity stress. Thus, eicosanoic was considered to play a possible signaling role. Its elevated levels in salinity-stressed plants may affect membrane fluidity [75]. Multiple studies have documented the role of fatty acids in regulating ion balance through voltage-gated ion channels [78]. The roots of both barley cultivars, sensitive and tolerant, experienced fast oxidation of membrane lipids during salinity stress. Differences in the oxidation pattern produced by salt are observed in both cultivars [75]. Hence, it can be suggested that there may be an interaction between sodium, potassium, and calcium ions, and unsaturated fatty acids, including oleic, linoleic, linolenic, and eicosanoic acids, and distinctive fatty acids such as cis-vaccenic, in the roots and seeds of plants.

## 5. Conclusions

Abiotic stress negatively impacts world agriculture. Salt disturbs the ecological equilibrium of the region and diminishes agricultural output. Elevated salinity reduces the rate at which plants absorb water. It modifies the physical and chemical characteristics of the soil. Salt diminishes crop yield, degrades soil fertility, and hampers economic production. Numerous morphological variations and metabolic reactions impact the germination of the seeds, plant growth, and water and nutrient intake, all affected by salty environments. Salt stress can stunt growth in the early stages of a plant’s life cycle. Because of this, agricultural output in salty areas falls, and plant products are worse in quality and quantity. This underscores the necessity of investigating the plant’s physiological reactions to severe circumstances using nanoparticle-based methods.

The present open-field study aimed to investigate the aggressive behavior of ZnO nanoparticles on maize plants when saline water fulfills the water needs during the growing season. This study sought to investigate the impact of applying saline water to the leaves on plants’ biochemistry, morphology, fatty acid production, and crop yield under both sodium chloride-free and sodium chloride-rich conditions. The results were analyzed regarding numerous morphological, biochemical, yield, and quality parameters. An evident decrease in traits and stages of plant development was observed in conjunction with salt stress. External application of ZnO-NPs to the leaves improved the salt stress tolerance of maize plants. Zinc oxide nanoparticles (ZnO-NPs) protect maize plants when exposed to salinity stress. They contribute to the protection of the cell membranes, increase the amount of chlorophyll, cause alterations in stress markers, strengthen the crop’s productivity and quality, and favorably impact the lipid composition. Thus, considering the characteristics of ZnO-NPs in maize plants under salt stress, it might be considered a feasible option for cultivating areas with restricted availability of high-quality irrigation water.

## Figures and Tables

**Table 1:** Quantification of chlorophyll levels (measured in SPAD units) in maize.

Treatments	Chlorophyll Levels at Various Growth Phases			
	20 June 2023	3 July 2023	15 July 2023	1 August 2023
**T1 Control (tap water)**	56.58 ± 0.27 ^ns^	60.54 ± 2.26 ^a^	64.00 ± 0.72 ^a^	63.62 ± 0.87 ^a^
**T2 (NaCl 150 mM)**	53.76 ± 0.20 ^ns^	49.11 ± 1.30 ^c^	50.66 ± 0.15 ^b^	50.58 ± 0.15 ^b^
**T3 (NaCl 150 mM + ZnO-NPs 2 g/L)**	56.26 ± 0.37 ^ns^	55.40 ± 0.64 ^b^	55.24 ± 1.53 ^b^	63.96 ± 1.05 ^a^
**T4 (tap water + ZnO-NPs 2 g/L)**	55.44 ± 1.97 ^ns^	58.40 ± 1.92 ^ab^	61.21 ± 2.39 ^a^	64.08 ± 0.90 ^a^
**LSD _(0.01)_**	ND	6.821	6.848	3.388
**LSD _(0.05)_**	ND	4.950	4.970	2.459
**Coefficient of variation**	4.093	6.610	6.416	3.027

The values indicate the mean is plus or minus the standard error. Superscripted letters indicate if the values are statistically distinguishable at a significance level of *p* < 0.05 or not. This comparison is made between various treatments within the same column. “^ns^”: non-significant. ND: non-determined.

**Table 2:** Measurement of plant height in maize plants.

Treatments	Plant Height (cm) at Various Growth Phases			
	20 June 2023	3 July 2023	15 July 2023	1 August 2023
**T1 Control (tap water)**	93.20 ± 0.41 ^b^	186.10 ± 2.76 ^b^	233.50 ± 1.87 ^b^	233.50 ± 1.87 ^b^
**T2 (NaCl 150 mM)**	80.23 ± 2.37 ^d^	162.64 ± 3.85 ^c^	208.43 ± 5.93 ^c^	209.71 ± 6.56 ^c^
**T3 (NaCl 150 mM + ZnO-NPs 2 g/L)**	86.00 ± 0.84 ^c^	191.60 ± 1.89 ^ab^	238.40 ± 1.44 ^ab^	238.80 ± 1.24 ^ab^
**T4 (tap water + ZnO-NPs 2 g/L)**	97.80 ± 1.55 ^a^	199.00 ± 1.52 ^a^	246.80 ± 1.36 ^a^	246.80 ± 1.36 ^a^
**LSD _(0.01)_**	6.154	10.891	13.471	14.590
**LSD _(0.05)_**	4.467	7.904	9.777	10.589
**Coefficient of variation**	3.730	3.189	3.146	3.401

The values indicate the mean is plus or minus the standard error. Superscripted letters indicate if the values are statistically distinguishable at a significance level of *p* < 0.05 or not. This comparison is made between various treatments within the same column.

**Table 3:** Measurement of stem diameter in maize plants.

Treatments	Stem Width (cm) at Various Growth Phases			
	20 June 2023	3 July 2023	15 July 2023	1 August 2023
**T1 Control (tap water)**	2.57 ± 0.07 ^a^	2.86 ± 0.09 ^a^	2.86 ± 0.09 ^a^	2.88 ± 0.08 ^bc^
**T2 (NaCl 150 mM)**	2.26 ± 0.05 ^b^	2.44 ± 0.12 ^b^	2.64 ± 0.09 ^b^	2.76 ± 0.02 ^c^
**T3 (NaCl 150 mM + ZnO-NPs 2 g/L)**	2.60 ± 0.03 ^a^	2.77 ± 0.05 ^a^	2.90 ± 0.00 ^a^	2.92 ± 0.02 ^b^
**T4 (tap water + ZnO-NPs 2 g/L)**	2.69 ± 0.07 ^a^	2.98 ± 0.00 ^a^	3.02 ± 0.04 ^a^	3.05 ± 0.03 ^a^
**LSD _(0.01)_**	0.226	0.359	0.266	0.173
**LSD _(0.05)_**	0.164	0.261	0.193	0.125
**Coefficient of variation**	4.831	7.040	5.045	3.220

The values indicate the mean is plus or minus the standard error. Superscripted letters indicate if the values are statistically distinguishable at a significance level of *p* < 0.05 or not. This comparison is made between various treatments within the same column.

**Table 4:** Measuring the leaf area in maize plants.

Treatments	Area of Leaves (cm^2^) at Various Growth Phases			
	20 June 2023	3 July 2023	15 July 2023	1 August 2023
**T1 Control (tap water)**	7271.28 ± 243.49 ^a^	9800.28 ± 201.28 ^b^	12,130.32 ± 275.13 ^a^	13,646.61 ± 309.52 ^a^
**T2 (NaCl 150 mM)**	5378.72 ± 135.50 ^c^	7389.00 ± 175.33 ^c^	10,296.00 ± 293.78 ^b^	11,668.80 ± 309.87 ^b^
**T3 (NaCl 150 mM + ZnO-NPs 2 g/L)**	6320.505 ± 205.30 ^b^	9441.18 ± 66.56 ^b^	12,348.33 ± 168.74 ^a^	13,963.05 ± 168.59 ^a^
**T4 (tap water + ZnO-NPs 2 g/L)**	6789.675 ± 58.62 ^ab^	10,447.215 ± 153.33 ^a^	12,411.6 ± 149.86 ^a^	14,495.22 ± 321.29 ^a^
**LSD _(0.01)_**	725.064	350.517	953.109	1174.721
**LSD _(0.05)_**	526.236	472.131	691.746	852.587
**Coefficient of variation**	6.113	3.810	4.396	4.751

The values indicate the mean is plus or minus the standard error. Superscripted letters indicate if the values are statistically distinguishable at a significance level of *p* < 0.05 or not. This comparison is made between various treatments within the same column.

**Table 5:** Analysis of biochemical and stress indicators in variously treated maize leaves.

Treatments	Concentrations/Treatments					
	Total Hydrolazable Sugars (mg/g)	Total Free Amino Acids (mg/g)	Protein Content (mg/g)	Proline Content (mg/g)	H_2_O_2_ (mg/g)	MDA (mmols/mL)
**T1 Control (tap water)**	177.50 ± 5.60 ^bc^	53.83 ± 1.23 ^c^	1.56 ± 0.03 ^b^	0.22 ± 0.00 ^b^	1.02 ± 0.01 ^d^	5.16 ± 0.46 ^c^
**T2 (NaCl 150 mM)**	228.49 ± 2.17 ^a^	74.35 ± 1.19 ^a^	0.93 ± 0.01 ^c^	0.32 ± 0.03 ^a^	1.78 ± 0.02 ^a^	13.06 ± 1.61 ^a^
**T3 (NaCl 150 mM + ZnO-NPs 2 g/L)**	186.31 ± 4.32 ^b^	56.71 ± 0.72 ^c^	0.98 ± 0.02 ^c^	0.31 ± 0.00 ^a^	1.35 ± 0.03 ^b^	9.35 ± 0.56 ^b^
**T4 (tap water + ZnO-NPs 2 g/L)**	170.76 ± 2.86 ^c^	60.83 ± 1.28 ^b^	2.39 ± 0.03 ^a^	0.23 ± 0.00 ^b^	1.19 ± 0.01 ^c^	5.97 ±0.41 ^c^
**LSD _(0.01)_**	17.136	4.861	0.491	3.206	0.086	3.913
**LSD _(0.05)_**	12.223	3.476	0.350	2.341	0.062	2.791
**Coefficient of variation**	4.158	3.663	16.112	2.938	2.985	21.594

The values indicate the mean is plus or minus the standard error. Superscripted letters indicate if the values are statistically distinguishable at a significance level of *p* < 0.05 or not. This comparison is made between various treatments within the same column.

**Table 6:** Post-harvest measurements from different treatments of maize.

Treatments	Weight of the Humid Leaves (g)	Weight of the Dried Leaves (g)	Weight of the Humid Stalk (g)	Weight of the Dried Stalk (g)	Weight of the Humid Cob (g)	Weight of Humid 100 Seeds (g)	Weight of Dried 100 Seeds (g)	Weight of Humid All Seeds (g)	Weight of Dried All Seeds (g)	Moisture in Corn Seeds (g)	No. of Rows/Cob	No. of Seeds/Row
**T1 Control (tap water)**	36.83 ± 1.17 ^a^	32.25 ± 0.81 ^b^	197.42 ± 8.38 ^bc^	73.22 ± 1.71 ^b^	314.98 ± 25.16 ^a^	40.00 ± 1.16 ^ab^	34.09 ± 1.20 ^bc^	239.85 ± 12.28 ^a^	204.20 ± 8.99 ^a^	0.15 ± 0.01 ^ns^	16 ± 0.33 ^ns^	40 ± 0.33 ^a^
**T2 (NaCl 150 mM)**	27.10 ± 0.78 ^b^	23.27 ± 1.02 ^c^	150.78 ± 14.58 ^c^	59.98 ± 1.86 ^b^	211.98 ± 13.75 ^b^	36.03 ± 2.53 ^b^	31.12 ± 1.32 ^c^	184.15 ± 11.78 ^b^	159.12 ± 5.64 ^b^	0.13 ± 0.02 ^ns^	16 ± 1.15 ^ns^	36 ± 1.86 ^b^
**T3 (NaCl 150 mM + ZnO-NPs 2 g/L)**	39.58 ± 1.28 ^a^	35.23 ± 0.59 ^a^	247.33 ± 21.53 ^ab^	115.88 ± 11.54 ^a^	301.57 ± 2.09 ^a^	43.58 ± 1.72 ^a^	38.46 ± 1.91 ^ab^	241.98 ± 11.44 ^a^	213.57 ± 12.16 ^a^	0.12 ± 0.01 ^ns^	16 ± 1.15 ^ns^	41 ± 1.33 ^a^
**T4 (tap water + ZnO-NPs 2 g/L)**	41.15 ± 1.20 ^a^	36.27 ± 0.95 ^a^	265.45 ± 29.18 ^a^	128.10 ± 8.44 ^a^	307.53 ± 3.50 ^a^	45.75 ± 2.63 ^a^	41.07 ± 1.48 ^a^	254.02 ± 1.06 ^a^	228.63 ± 5.92 ^a^	0.10 ± 0.02 ^ns^	15 ± 0.67 ^ns^	43 ± 0.33 ^a^
**LSD _(0.01)_**	6.552	4.078	ND	34.445	68.712	ND	7.124	48.699	40.776	ND	ND	ND
**LSD _(0.05)_**	4.503	2.803	65.185	23.675	47.228	6.845	4.897	33.473	28.027	ND	ND	3.805
**Coefficient of variation**	6.659	4.688	16.084	13.035	8.832	8.793	7.187	7.729	7.392	25.784	9.767	5.031

The values indicate the mean is plus or minus the standard error. Superscripted letters indicate if the values are statistically distinguishable at a significance level of *p* < 0.05 or not. This comparison is made between various treatments within the same column. ND: non-determined. “^ns^”: non-significant.

**Table 7:** Changes in dry matter, crude protein, ADF, and NDF values in the leaves of maize.

Treatments	Concentrations (g/100 g Dry Weight)			
	Dry Matter	Crude Protein	Acid Detergent Fiber (ADF)	Neutral Detergent Fiber (NDF)
**T1 Control (tap water)**	78.83 ± 0.41 ^ns^	6.87 ± 0.00 ^b^	21.17 ± 0.20 ^a^	34.53 ± 0.49 ^a^
**T2 (NaCl 150 mM)**	78.08 ± 0.70 ^ns^	6.05 ± 0.23 ^c^	19.82 ± 0.02 ^b^	31.42 ± 0.11 ^b^
**T3 (NaCl 150 mM + ZnO-NPs 2 g/L)**	78.56 ± 0.73 ^ns^	7.26 ± 0.08 ^a^	20.21 ± 0.33 ^b^	32.53 ± 0.73 ^b^
**T4 (tap water + ZnO-NPs 2 g/L)**	78.92 ± 1.01 ^ns^	7.35 ± 0.02 ^a^	21.57 ± 0.37 ^a^	34.60 ± 0.48 ^a^
**LSD _(0.01)_**	ND	0.539	1.140	2.181
**LSD _(0.05)_**	ND	0.384	0.813	1.555
**Coefficient of variation**	1.894	3.624	2.551	3.034

The values indicate the mean is plus or minus the standard error. Superscripted letters indicate if the values are statistically distinguishable at a significance level of *p* < 0.05 or not. This comparison is made between various treatments within the same column. ND: non-determined. “^ns^”: non-significant.

**Table 8:** Changes in moisture, crude protein, crude fat, and starch values in the grains of maize.

Treatments	Concentrations (g/100 g Dry Weight)			
	Moisture	Crude Protein	Crude Fat	Starch
**T1 Control (tap water)**	5.58 ± 0.09 ^d^	8.57 ± 0.15 ^b^	3.60 ± 0.02 ^ns^	68.84 ± 0.03 ^b^
**T2 (NaCl 150 mM)**	6.77 ± 0.12 ^b^	7.43 ± 0.06 ^c^	3.64 ± 0.04 ^ns^	68.21 ± 0.04 ^c^
**T3 (NaCl 150 mM + ZnO-NPs 2 g/L)**	7.07 ± 0.05 ^a^	7.79 ± 0.25 ^c^	3.54 ± 0.01 ^ns^	69.55 ± 0.12 ^a^
**T4 (tap water + ZnO-NPs 2 g/L)**	6.40 ±0.07 ^c^	9.20 ± 0.17 ^a^	3.60 ± 0.02 ^ns^	68.19 ± 0.02 ^c^
**LSD _(0.01)_**	0.383	0.730	ND	0.299
**LSD _(0.05)_**	0.273	0.521	ND	0.2131
**Coefficient of variation**	2.749	4.101	1.331	0.201

The values indicate the mean is plus or minus the standard error. Superscripted letters indicate if the values are statistically distinguishable at a significance level of *p* < 0.05 or not. This comparison is made between various treatments within the same column. ND: non-determined. “^ns^”: non-significant.

**Table 9:** Gas chromatographic (GC) analysis of long-chain fatty acids (LCFA) in maize grains.

Compounds to Be Detected	Concentrations (g/100 g)/Treatments			
	T1	T2	T3	T4
Myristic acid	0.04	0.07	0.06	0.06
Palmitic acid	13.32	13.66	12.9	13.3
Palmitoleic acid	0.05	0.05	0.05	0.04
Stearic acid	2.43	2.67	2.62	2.54
Oleic acid	27.09	27.84	27.76	27.53
Vaccenic acid	0.34	0.30	0.32	0.33
Linoleic acid	50.54	51.79	51.29	51.09
γ-linolenic acid	0.05	0.06	0.07	0.05
α-linolenic acid	0.93	0.95	0.95	0.7
Ecosenoic acid	0.10	0.13	0.11	0.10
Arachidonic acid	0.03	0.03	0.03	0.02
Eicosapentaenoic acid (EPA)	0	0	0	0
Docosadienoic acid	0	0	0	0
Docosapentaenoic acid	0	0	0	0
Docosahexaenoic acid (DHA)	0.02	0.01	0.02	0.02
Total	94.94	97.56	96.00	95.78

T1: control (tap water); T2: 150 mM sodium chloride; T3: 150 mM sodium chloride + 2 g/L ZnO-NPs; and T4: tap water + 2 g/L ZnO-NPs.

## Data Availability

Data are contained within this article.

## Appendix A

**Table A1:** The total volume of water (L) applied from the initiation of sowing maize in different treatments until the harvest.

Day after Sowing	Month	WR for Each Plant (mm/Specific Period)	WR for One Plant (L/Specific Period)	WR for One Subplot (L/Specific Period)	Water is Needed for the Whole Day of Irrigation
**1–35**	1 May–4 June	The plants left for rainfall irrigation during the first three weeks			
Volume of required water at each period = Depth of water (m) × Area needed to be irrigated (m^2^) [79] Notes: Plant density was supposed to be 12 plant/1 m^2^ The area occupied by one plant is 0.3 × 0.3 = 0.09 m^2^ Each subplot contains 3 rows and each row contains 15 plants, so the subplot contains 45 plants					
**36–46**	5–15 June	10.4 mm	0.936 mL	0.936 × 45 = 42.12 L	336.96 L for saline and the same for non-saline treatments = 673.92 L/day
**47–57**	15–25 June	21.1 mm	1.89 L	1.89 × 45 = 85.45	683.64 L for saline and the same for non-saline = 1367.28 L/day
**58–68**	25 June–5 July	34.00 mm	3.06 L	3.06 × 45 = 137.7	1106.6 L for saline and the same for non-saline treatments = 2123.2 L/day
**69–79**	5–15 July	41.8 mm	3.06 L	3.06 × 45 = 137.7	1106.6 L for saline and the same for non-saline treatments = 2123.2 L/day
**80–90**	15–25 July	45.00 mm	3.06 L	3.06 × 45 = 137.7	1106.6 L for saline and the same for non-saline treatments = 2123.2 L/day
**91–101**	25 July–4 August	45.2 mm	3.06 L	3.06 × 45 = 137.7	1106.6 L for saline and the same for non-saline treatments = 2123.2 L/day
**102–112**	4–14 August	45.2 mm	3.06 L	3.06 × 45 = 137.7	1106.6 L for saline and the same for non-saline treatments = 2123.2 L/day
**113–123**	14–24 August	45.2 mm	3.06 L	3.06 × 45 = 137.7	1106.6 L for saline and the same for non-saline treatments = 2123.2 L/day
**124–134**	24 August–2 September	45.2 mm	3.06 L	3.06 × 45 = 137.7	1106.6 L for saline and the same for non-saline treatments = 2123.2 L/day

## References

[1] Ahmed M., Tóth Z., Decsi K. (2024). The Impact of Salinity on Crop Yields and the Confrontational Behavior of Transcriptional Regulators, Nanoparticles, and Antioxidant Defensive Mechanisms under Stressful Conditions: A Review. Int. J. Mol. Sci..

[2] Atta K., Mondal S., Gorai S., Singh A.P., Kumari A., Ghosh T., Roy A., Hembram S., Gaikwad D.J., Mondal S. (2023). Impacts of Salinity Stress on Crop Plants: Improving Salt Tolerance through Genetic and Molecular Dissection. Front. Plant Sci..

[3] Gul Z., Tang Z.-H., Arif M., Ye Z. (2022). An Insight into Abiotic Stress and Influx Tolerance Mechanisms in Plants to Cope in Saline Environments. Biology.

[4] Mukhopadhyay R., Sarkar B., Jat H.S., Sharma P.C., Bolan N.S. (2021). Soil Salinity under Climate Change: Challenges for Sustainable Agriculture and Food Security. J. Environ. Manag..

[5] Akmal M.A., Mirzakamol A., Mukhtor M.D., Sardor N., Akramjon M., Ilhomjon B., Naim N.K., Ildiko M., Zabardast T.B., Ibrokhim Y.A. (2023). Chemical Interventions to Alleviate Salt Stress in Cotton Plants: A Review. Plant Sci. Today.

[6] Sah R.P., Chakraborty M., Prasad K., Pandit M., Tudu V.K., Chakravarty M.K., Narayan S.C., Rana M., Moharana D. (2020). Impact of Water Deficit Stress in Maize: Phenology and Yield Components. Sci. Rep..

[7] Pandit M., Chakraborty M., Haider Z.A., Pande A., Sah R.P., Sourav K. (2016). Genetic Diversity Assay of Maize (*Zea mays* L.) Inbreds Based on Morphometric Traits and SSR Markers. AJAR.

[8] Chaudhary D., Kumar A., Kumar R., Singode A., Mukri G., Sah R., Tiwana U., Kumar B. (2016). Evaluation of Normal and Specialty Corn for Fodder Yield and Quality Traits. Range Manag. Agrofor..

[9] Sah R., Ahmed S., Malaviya D., Saxena P. (2016). Identification of Consistence Performing Dual Purpose Maize (*Zea mays* L.) Genotypes under Semi-Arid Condition. Range Manag. Agrofor..

[10] Manigopa C., Sah R. (2012). Genetic Component in Baby Corn (*Zea mays* L.). Plant Arch..

[11] FAOStat (2021). FAO Stat.

[12] Ren X., Jia Z., Chen X. (2008). Rainfall Concentration for Increasing Corn Production under Semiarid Climate. Agric. Water Manag..

[13] Ahmed M., Decsi K., Tóth Z. (2022). Different Tactics of Synthesized Zinc Oxide Nanoparticles, Homeostasis Ions, and Phytohormones as Regulators and Adaptatively Parameters to Alleviate the Adverse Effects of Salinity Stress on Plants. Life.

[14] Ahmed M., Marrez D.A., Abdelmoeen N.M., Mahmoud E.A., Abdel-Shakur Ali M., Decsi K., Tóth Z. (2023). Proximate Analysis of Moringa Oleifera Leaves and the Antimicrobial Activities of Successive Leaf Ethanolic and Aqueous Extracts Compared with Green Chemically Synthesized Ag-NPs and Crude Aqueous Extract against Some Pathogens. Int. J. Mol. Sci..

[15] Ahmed M., Marrez D.A., Mohamed Abdelmoeen N., Abdelmoneem Mahmoud E., Ali M.A.-S., Decsi K., Tóth Z. (2023). Studying the Antioxidant and the Antimicrobial Activities of Leaf Successive Extracts Compared to the Green-Chemically Synthesized Silver Nanoparticles and the Crude Aqueous Extract from Azadirachta Indica. Processes.

[16] Seleiman M.F., Ahmad A., Battaglia M.L., Bilal H.M., Alhammad B.A., Khan N. (2023). Zinc Oxide Nanoparticles: A Unique Saline Stress Mitigator with the Potential to Increase Future Crop Production. South Afr. J. Bot..

[17] Solaiman M.A., Ali M.A., Abdel-Moein N.M., Mahmoud E.A. (2020). Synthesis of Ag-NPs Developed by Green-Chemically Method and Evaluation of Antioxidant Activities and Anti-Inflammatory of Synthesized Nanoparticles against LPS-Induced NO in RAW 264.7 Macrophages. Biocatal. Agric. Biotechnol..

[18] Alabdallah N.M., Alzahrani H.S. (2020). The Potential Mitigation Effect of ZnO Nanoparticles on [*Abelmoschus esculentus* L. Moench] Metabolism under Salt Stress Conditions. Saudi J. Biol. Sci..

[19] Ahmed M., Marrez D.A., Rizk R., Zedan M., Abdul-Hamid D., Decsi K., Kovács G.P., Tóth Z. (2024). The Influence of Zinc Oxide Nanoparticles and Salt Stress on the Morphological and Some Biochemical Characteristics of *Solanum lycopersicum* L. Plants. Plants.

[20] Faizan M., Bhat J.A., Chen C., Alyemeni M.N., Wijaya L., Ahmad P., Yu F. (2021). Zinc Oxide Nanoparticles (ZnO-NPs) Induce Salt Tolerance by Improving the Antioxidant System and Photosynthetic Machinery in Tomato. Plant Physiol. Biochem..

[21] Food and Agriculture Organization (1998). Crop Evapo-Transpiration—Guidelines for Computing Crop Water Requirements—FAO Irrigation and Drainage Paper 56.

[22] Nejati K., Rezvani Z., Pakizevand R. (2011). Synthesis of ZnO Nanoparticles and Investigation of the Ionic Template Effect on Their Size and Shape. Int. Nano Lett..

[23] Sakalova G.V. (1979). Environment and Experimental of Plant Growth.

[24] Musa U., Hassan U. (2016). Leaf Area Determination for Maize (*Zea mays* L.), Okra (*Abelmoschus esculentus* L.) and Cowpea (*Vigna unguiculata* L.) Crops using Linear Measurements. J. Biol. Agric. Heal..

[25] Bates L.S., Waldren R.P., Teare I.D. (1973). Rapid Determination of Free Proline for Water-Stress Studies. Plant Soil.

[26] Bradford M.M. (1976). A Rapid and Sensitive Method for the Quantitation of Microgram Quantities of Protein Utilizing the Principle of Protein-Dye Binding. Anal. Biochem..

[27] Prud’homme M.-P., Gonzalez B., Billard J.-P., Boucaud J. (1992). Carbohydrate Content, Fructan and Sucrose Enzyme Activities in Roots, Stubble and Leaves of Ryegrass (*Lolium perenne* L.) as Affected by Source/Sink Modification after Cutting. J. Plant Physiol..

[28] Heath R.L., Packer L. (1968). Photoperoxidation in Isolated Chloroplasts. I. Kinetics and Stoichiometry of Fatty Acid Peroxidation. Arch. Biochem. Biophys..

[29] Alexieva V., Sergiev I., Mapelli S., Karanov E. (2001). The Effect of Drought and Ultraviolet Radiation on Growth and Stress Markers in Pea and Wheat. Plant Cell Environ..

[30] Cequier-Sánchez E., Rodríguez C., Ravelo Á.G., Zárate R. (2008). Dichloromethane as a Solvent for Lipid Extraction and Assessment of Lipid Classes and Fatty Acids from Samples of Different Natures. J. Agric. Food Chem..

[31] (2015). Animal and Vegetable Fats and Oils-Gas Chromatography of Fatty Acid Methyl Esters. Part 1. Guidelines on Modern Gas Chromatography of Fatty Acid Methyl Esters.

[32] Stępień A., Wojtkowiak K., Kolankowska E., Pietrzak-Fiećko R. (2024). Corn Grain Fatty Acid Contents in Response to Organic Fertilisers from Meat Industry Waste. Sustainability.

[33] Van Soest P.J., Robertson J.B., Lewis B.A. (1991). Methods for Dietary Fiber, Neutral Detergent Fiber, and Nonstarch Polysaccharides in Relation to Animal Nutrition. J. Dairy Sci..

[34] AOAC (2019). Official Methods of Analysis of the Association of Official Analytical Chemists: Official Methods of Analysis of AOAC International.

[35] Jangam A.K., Thali P. (2004). WASP-Web Agri Stat Package.

[36] Maher S., Nisar S., Aslam S.M., Saleem F., Behlil F., Imran M., Assiri M.A., Nouroz A., Naheed N., Khan Z.A. (2023). Synthesis and Characterization of ZnO Nanoparticles Derived from Biomass (*Sisymbrium irio*) and Assessment of Potential Anticancer Activity. ACS Omega.

[37] Mahamuni P.P., Patil P.M., Dhanavade M.J., Badiger M.V., Shadija P.G., Lokhande A.C., Bohara R.A. (2019). Synthesis and Characterization of Zinc Oxide Nanoparticles by Using Polyol Chemistry for Their Antimicrobial and Antibiofilm Activity. Biochem. Biophys. Rep..

[38] Amin Z.S., Afzal M., Ahmad J., Ahmed N., Zeshan B., Hashim N.H.H.N., Yean C.Y. (2023). Synthesis, Characterization and Biological Activities of Zinc Oxide Nanoparticles Derived from Secondary Metabolites of Lentinula Edodes. Molecules.

[39] Badawy S.A., Zayed B.A., Bassiouni S.M.A., Mahdi A.H.A., Majrashi A., Ali E.F., Seleiman M.F. (2021). Influence of Nano Silicon and Nano Selenium on Root Characters, Growth, Ion Selectivity, Yield, and Yield Components of Rice (*Oryza sativa* L.) under Salinity Conditions. Plants.

[40] Gerona M.E.B., Deocampo M.P., Egdane J.A., Ismail A.M., Dionisio-Sese M.L. (2019). Physiological Responses of Contrasting Rice Genotypes to Salt Stress at Reproductive Stage. Rice Sci..

[41] Ahmad A., Tola E., Alshahrani T.S., Seleiman M.F. (2023). Enhancement of Morphological and Physiological Performance of *Zea mays* L. under Saline Stress Using ZnO Nanoparticles and 24-Epibrassinolide Seed Priming. Agronomy.

[42] Yang Z., Li J.-L., Liu L.-N., Xie Q., Sui N. (2020). Photosynthetic Regulation Under Salt Stress and Salt-Tolerance Mechanism of Sweet Sorghum. Front. Plant Sci..

[43] Seleiman M.F., Al-Selwey W.A., Ibrahim A.A., Shady M., Alsadon A.A. (2023). Foliar Applications of ZnO and SiO_2_ Nanoparticles Mitigate Water Deficit and Enhance Potato Yield and Quality Traits. Agronomy.

[44] Cakmak I. (2008). Enrichment of Cereal Grains with Zinc: Agronomic or Genetic Biofortification?. Plant Soil.

[45] El-Badri A.M., Batool M., Mohamed I.A., Khatab A., Sherif A., Wang Z. (2021). Modulation of Salinity Impact on Early Seedling Stage via Nano-Priming Application of Zinc Oxide on Rapeseed (*Brassica napus* L.). Plant Physiol. Biochem..

[46] Anjum N.A., Gill S.S., Corpas F.J., Ortega-Villasante C., Hernandez L.E., Tuteja N., Sofo A., Hasanuzzaman M., Fujita M. (2022). Editorial: Recent Insights Into the Double Role of Hydrogen Peroxide in Plants. Front. Plant Sci..

[47] Naseer I., Javad S., Iqbal S., Shah A.A., Alwutayd K., AbdElgawad H. (2023). Deciphering the Role of Zinc Oxide Nanoparticles on Physiochemical Attributes of *Zea mays* Exposed to Saline Conditions through Modulation in Antioxidant Enzyme Defensive System. South Afr. J. Bot..

[48] Raliya R., Tarafdar J.C. (2013). ZnO Nanoparticle Biosynthesis and Its Effect on Phosphorous-Mobilizing Enzyme Secretion and Gum Contents in Clusterbean (*Cyamopsis tetragonoloba* L.). Agric. Res..

[49] Mahawar L., Živčák M., Barboricova M., Kovár M., Filaček A., Ferencova J., Vysoká D.M., Brestič M. (2024). Effect of Copper Oxide and Zinc Oxide Nanoparticles on Photosynthesis and Physiology of *Raphanus sativus* L. under Salinity Stress. Plant Physiol. Biochem..

[50] Rai-Kalal P., Jajoo A. (2021). Priming with Zinc Oxide Nanoparticles Improve Germination and Photosynthetic Performance in Wheat. Plant Physiol. Biochem..

[51] Tavanti T.R., Melo A.A.R.D., Moreira L.D.K., Sanchez D.E.J., Silva R.D.S., Silva R.M.D., Reis A.R.D. (2021). Micronutrient Fertilization Enhances ROS Scavenging System for Alleviation of Abiotic Stresses in Plants. Plant Physiol. Biochem..

[52] Mogazy A.M., Hanafy R.S. (2022). Foliar Spray of Biosynthesized Zinc Oxide Nanoparticles Alleviate Salinity Stress Effect on Vicia Faba Plants. J. Soil Sci. Plant Nutr..

[53] Raddy R., Salimath M., Geetha K.N., Shankar A. (2018). ZnO Nanoparticle Improves Maize Growth, Yield and Seed Zinc under High Soil pH Condition. Int. J. Curr. Microbiol. Appl. Sci..

[54] Tondey M., Kalia A., Singh A., Dheri G.S., Taggar M.S., Nepovimova E., Krejcar O., Kuca K. (2021). Seed Priming and Coating by Nano-Scale Zinc Oxide Particles Improved Vegetative Growth, Yield and Quality of Fodder Maize (*Zea mays*). Agronomy.

[55] Satdev V.J., Chavda B.N., Saini L.K. (2020). Effect of Nano ZnO on Growth and Yield of Sweet Corn under South Gujarat Condition. Int. J. Chem. Stud..

[56] Zhou D., Jin S., Li L., Wang Y., Weng N. (2011). Quantifying the Adsorption and Uptake of CuO Nanoparticles by Wheat Root Based on Chemical Extractions. J. Environ. Sci..

[57] Lin D., Xing B. (2008). Root Uptake and Phytotoxicity of ZnO Nanoparticles. Environ. Sci. Technol..

[58] Vago M.E., Jaurena G., Estevez J.M., Castro M.A., Zavala J.A., Ciancia M. (2021). Salt Stress on *Lotus tenuis* Triggers Cell Wall Polysaccharide Changes Affecting Their Digestibility by Ruminants. Plant Physiol. Biochem..

[59] Beauchemin K.A. (1996). Using ADF and NDF in Dairy Cattle Diet Formulation—A Western Canadian Perspective. Anim. Feed Sci. Technol..

[60] Boga M., Yurtseven S., Kilic U., Aydemir S., Polat T. (2014). Determination of Nutrient Contents and in Vitro Gas Production Values of Some Legume Forages Grown in the Harran Plain Saline Soils. Asian Australas. J. Anim. Sci..

[61] Ben-Ghedalia D., Solomon R., Miron J., Yosef E., Zomberg Z., Zukerman E., Greenberg A., Kipnis T. (2001). Effect of Water Salinity on the Composition and in Vitro Digestibility of Winter-Annual Ryegrass Grown in the Arava Desert. Anim. Feed Sci. Technol..

[62] De Lima R.B., Dos Santos T.B., Vieira L.G.E., De Lourdes Lúcio Ferrarese M., Ferrarese-Filho O., Donatti L., Boeger M.R.T., De Oliveira Petkowicz C.L. (2014). Salt Stress Alters the Cell Wall Polysaccharides and Anatomy of Coffee (*Coffea arabica* L.) Leaf Cells. Carbohydr. Polym..

[63] Kerepesi I., Galiba G. (2000). Osmotic and Salt Stress-Induced Alteration in Soluble Carbohydrate Content in Wheat Seedlings. Crop Sci..

[64] Khatkar D., Kuhad M.S. (2000). Short-Term Salinity Induced Changes in Two Wheat Cultivars at Different Growth Stages. Biol. Plant..

[65] Sabagh A.E., Hossain A., Iqbal M.A., Barutçular C., Islam M.S., Çiğ F., Erman M., Sytar O., Brestic M., Wasaya A. (2020). Maize Adaptability to Heat Stress under Changing Climate. Plant Stress Physiology.

[66] Weinberg Z.G., Yan Y., Chen Y., Finkelman S., Ashbell G., Navarro S. (2008). The Effect of Moisture Level on High-Moisture Maize (*Zea mays* L.) under Hermetic Storage Conditions—In Vitro Studies. J. Stored Prod. Res..

[67] Li J., Chen J., Jin J., Wang S., Du B. (2019). Effects of Irrigation Water Salinity on Maize (*Zea may* L.) Emergence, Growth, Yield, Quality, and Soil Salt. Water.

[68] Kang Y., Wan S. (2005). Effect of Soil Water Potential on Radish (*Raphanus sativus* L.) Growth and Water Use under Drip Irrigation. Sci. Hortic..

[69] Gogna M., Choudhary A., Mishra G., Kapoor R., Bhatla S.C. (2020). Changes in Lipid Composition in Response to Salt Stress and Its Possible Interaction with Intracellular Na+-K+ Ratio in Sunflower (*Helianthus annuus* L.). Environ. Exp. Bot..

[70] Kumar D., Yusuf M.A., Singh P., Sardar M., Sarin N.B. (2013). Modulation of Antioxidant Machinery in α-Tocopherol-Enriched Transgenic Brassica Juncea Plants Tolerant to Abiotic Stress Conditions. Protoplasma.

[71] Noreen S., Ashraf M. (2010). Modulation of Salt (NaCl)-Induced Effects on Oil Composition and Fatty Acid Profile of Sunflower (*Helianthus annuus* L.) by Exogenous Application of Salicylic Acid. J. Sci. Food Agric..

[72] Di Caterina R., Giuliani M.M., Rotunno T., De Caro A., Flagella Z. (2007). Influence of Salt Stress on Seed Yield and Oil Quality of Two Sunflower Hybrids. Ann. Appl. Biol..

[73] Hong Y., Zhao J., Guo L., Kim S.-C., Deng X., Wang G., Zhang G., Li M., Wang X. (2016). Plant Phospholipases D and C and Their Diverse Functions in Stress Responses. Prog. Lipid Res..

[74] Liu X., Ma D., Zhang Z., Wang S., Du S., Deng X., Yin L. (2019). Plant Lipid Remodeling in Response to Abiotic Stresses. Environ. Exp. Bot..

[75] Yu D., Boughton B.A., Hill C.B., Feussner I., Roessner U., Rupasinghe T.W.T. (2020). Insights Into Oxidized Lipid Modification in Barley Roots as an Adaptation Mechanism to Salinity Stress. Front. Plant Sci..

[76] Yu B.J., Gong H.M., Liu Y.L. (1999). Effects of Exogenous Fatty Acids on H+-ATPase Activity and Lipid Composition of Plasma Membrane Vesicles Isolated from Roots of Barley Seedlings under Salt Stress. J. Plant Physiol..

[77] Paulucci N.S., Medeot D.B., Dardanelli M.S., De Lema M.G. (2011). Growth Temperature and Salinity Impact Fatty Acid Composition and Degree of Unsaturation in Peanut-Nodulating Rhizobia. Lipids.

[78] Elinder F., Liin S.I. (2017). Actions and Mechanisms of Polyunsaturated Fatty Acids on Voltage-Gated Ion Channels. Front. Physiol..

[79] Doorenbos J., Pruitt W.O. (1977). Crop Water Requirements. FAO Irrigation and Drainage Paper 24.

